# Spatial Evolution of Antimicrobial Metallopolymers toward the Main Chain Topology

**DOI:** 10.1021/acscentsci.6c00694

**Published:** 2026-06-19

**Authors:** Md Waliullah Hossain, Marsh Kral, Aylin Feuerstein, Swagatam Barman, Alimi Abiodun, Markus Gallei, Chao Cai, André Schäfer, Chuanbing Tang

**Affiliations:** † Department of Chemistry and Biochemistry, 2629 University of South Carolina, Columbia, South Carolina 29208, United States; ‡ Department of Chemistry, Faculty of Natural Sciences and Technology, Saarland University, 66123 Saarbrücken, Germany; § Saarland Center for Energy Materials and Sustainability - Saarene, Saarland University, 66123 Saarbrücken, Germany; ∥ Clinical Pharmacy and Outcomes Sciences (CPOS), College of Pharmacy, University of South Carolina, Columbia, South Carolina 29208, United States

## Abstract

Synthetic polymers and peptides with spatial control have emerged as effective molecular agents for addressing antimicrobial resistance (AMR). We report on one of the first kinds of studies on how spatial compositions in cationic metallopolymers can be tuned to control their antimicrobial efficacy. As a representative, the evolution of cobaltocenium cation from side chain to main chain has shown drastic impacts on polymer–cell interactions and consequential killing of microbes. A main-chain cobaltocenium polymer exhibited broad-spectrum activity against multidrug-resistant (MDR) bacteria, primarily targeting bacterial cell membranes and outer leaflets. The polymer also showed a strong efficacy in treating mature biofilms across multiple bacterial strains. Resistance development was evaluated over 20 passages, with no detectable resistance. Overall, these findings reveal spatial control of cationic centers in metallopolymers as a unique molecular platform for combating MDR pathogens and alleviating the AMR burden on global healthcare settings.

## Graphical abstract

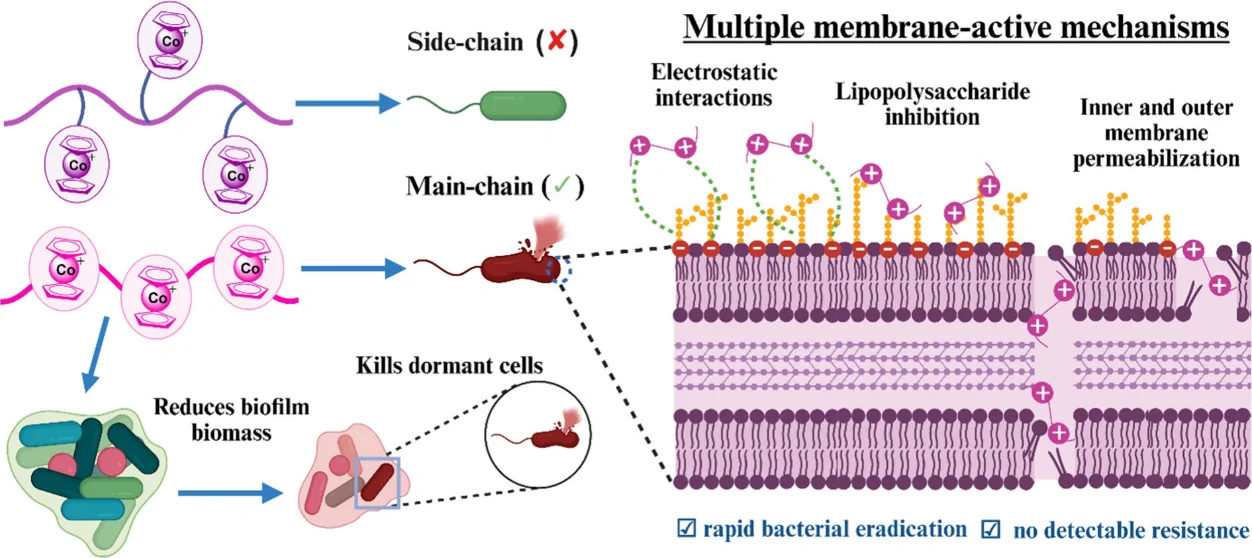

## Introduction

According to the World Health Organization, antimicrobial resistance (AMR) poses a serious threat to the public health system worldwide.^1^ There has been a severe dearth of new classes of antibiotics, with a variety of existing ones becoming essentially inactive due to the rise of AMR. In the past 25 years, only six antibiotics have reached clinical settings; however, none of them are active against Gram-negative bacteria.^2^ Antibiotics work in a “lock and key” method by targeting specific metabolic pathways of a bacterial cell. In response, bacteria can evolve through various molecular mechanisms to become resistant to antibiotics.^3,4^

Nature has used its own arsenal of antimicrobial agents, known as antimicrobial peptides (AMPs), as part of the innate immune system of multicellular eukaryotes.^5^ AMPs are short-chain amphipathic proteins with a broad-spectrum activity against pathogens. They have several modes of action, which can disrupt different metabolic processes of pathogens with membrane disruption being one of the major ones.^6^ Additionally, some AMPs can translocate through bacterial membranes and bind to proteins and nucleic acids.^7^ Although they are potent, their applicability is limited due to low bioavailability, proteolytic instability, high synthetic cost, and cytotoxicity.^8,9^ The design principle of AMPs necessitates spatial control of cationic and hydrophobic residues in order to provide selectivity toward microbes.^10^ To date, several synthetic polymers and peptides have been developed by mimicking the structure and function of AMPs to combat microbial infection.^11–19^

There have been tremendous efforts in synthesizing biomacromolecules by controlling cations, hydrophilic and hydrophobic residues, molecular weight, etc.^20,21^ Spatial control on synthetic AMP-mimicking polymers also plays a critical role in inducing lysis or inhibiting the growth of bacteria.^22–27^ The arrangement of cations can be categorized into two major classes of polymers: side-chain vs main-chain. Compared to a side-chain molecular architecture, placing cations in the main-chain is hypothesized to have the following benefits: a) When the cation is part of the backbone, it is harder for the polymer to “lose” its cationic functionality through side-chain hydrolysis or linker cleavage. This can be an advantage for long-term contact-killing surfaces with reduced leachability. b) Backbone charges can give a regular spacing of cations with less conformational “hiding” of charges compared with flexible side chains. It can improve the initial binding/adsorption rate to bacterial membranes. c) Main-chain cations can enforce preferred conformations for surface alignment to promote “carpet-like” coverage and increase the likelihood of cooperative disruption.

A few studies are listed here to illustrate the effects of spatial control on antimicrobial polymers (Figure 1). Yang and co-workers developed polyionenes by incorporating quaternary ammonium into the polymer backbone with variable linkers to tune amphiphilicity, which exhibited significant improvement in antimicrobial activity.^28^ Yan and co-workers carried out a systematic study to compare side-chain and main-chain polymers with different nitrogen-atom bearing cations and showed improved efficacy in main-chain type polymer systems.^29^ The Khan group reported sulfonium containing main-chain polymers with better activity than side-chain sulfoniums,^30^ and concurrently the Rao group reported main-chain polysulfoniums with an alternating hydrophobic sequence for better activity and biocompatibility.^31^ The Feng group developed main-chain oligoamidine polymers which are dual targeting, i.e., DNA binding and membrane-active.^32^ Similarly, the Bai group developed oligoguanidine with alternating amphiphilicity by placing the cation and hydrophobic group both in the main chain of the polymer backbone with the same dual mechanism of action.^33^ Chan-Park and co-workers have developed main-chain containing polymers and showed that placing the cation into the main chain with tuned amphiphilicity can result in significant improvement of antimicrobial activity.^34–36^

**Figure 1:**
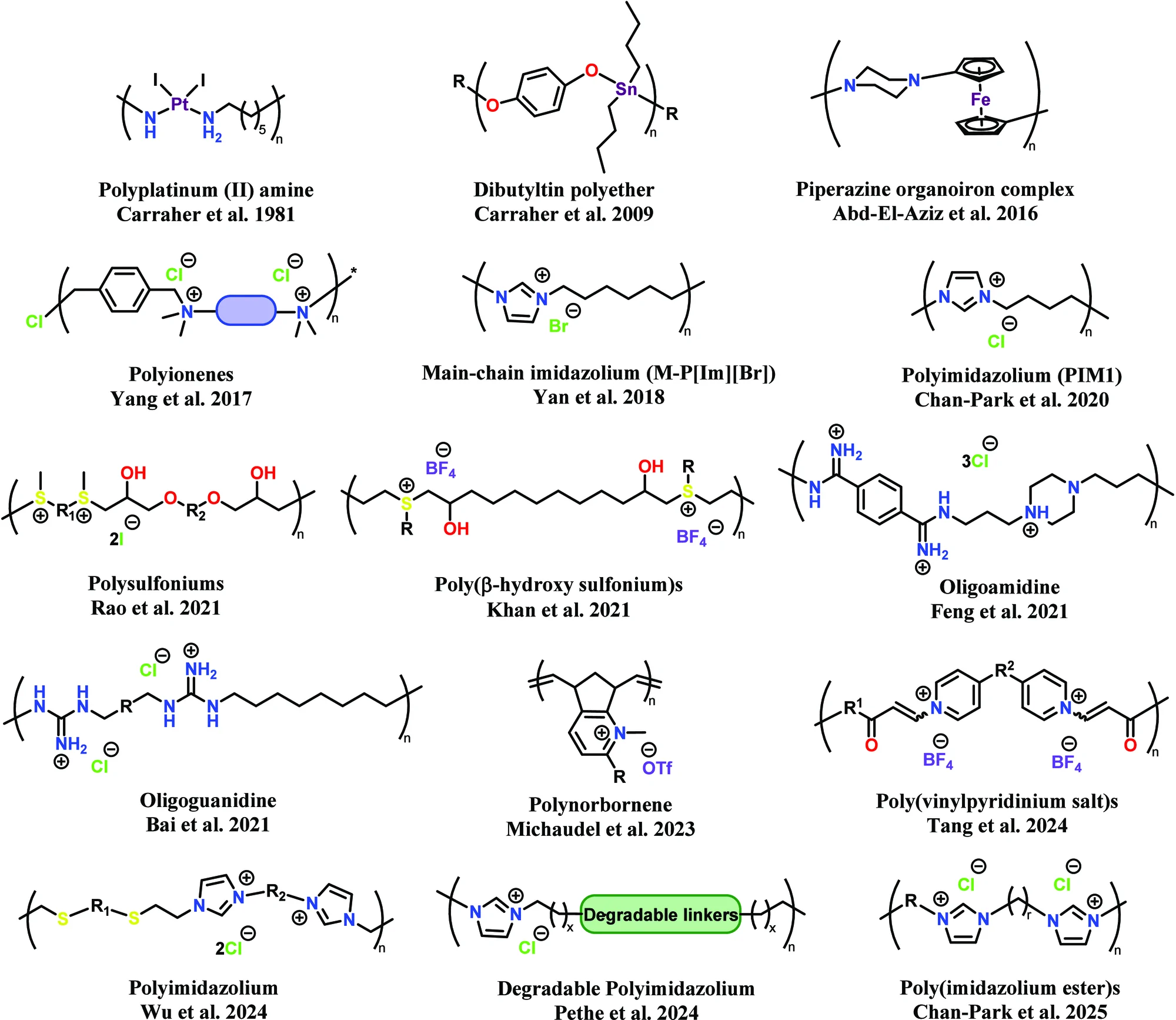
Representative main-chain cationic antimicrobial polymers.

There is a lack of research involving the spatial arrangement of cationic centers as a key design parameter in AMP-mimicking metallopolymers. It has been demonstrated earlier that placing the cation into the polymer backbone is beneficial compared to the side chain.^29^ Alongside this, metallopolymers have shown excellent promise in the field of antimicrobial research, due to the prevalence of metal-based cations.^37–42^ Spatial control of metal cations or organometallic cations is rare in developing antimicrobial polymers.^43–45^ Cationic cobaltocenium is a unique cation, with its superior stability against a variety of pH conditions and its soft nature compared to small quaternary ammoniums.^46–48^ Cobaltocenium has been successfully integrated into a polymeric framework for antimicrobial design, where cobaltocenium can combine with antibiotics to resensitize different classes of antibiotics against both Gram-positive and Gram-negative bacteria.^49–51^ Previously, polymers containing both cobaltocenium and primary ammonium have shown antimicrobial efficacy with no detectable resistance against Gram-negative bacteria.^52^ However, all of these polymers with cobaltocenium at the side-chain showed low antimicrobial efficacy on their own.

Herein, we report the first main-chain cobaltocenium-containing polymer as a robust antimicrobial agent that exhibits high antimicrobial efficacy compared to its side-chain counterpart. While there are main-chain antimicrobial metallopolymers reported, such as those using metal–ligand complexes,^53^ to our knowledge, cobaltocenium would be the first covalent organometallic building blocks in the main chain.

## Results and Discussion

### Synthesis of Cobaltocenium-Containing Polymers

To evaluate the spatial effect, we synthesized two polymer systems: one with the cobaltocenium cation in the main chain and the other in the side chain. It is worth mentioning that the synthesis of water-soluble main-chain cobaltocenium-containing polymers has been challenging. Among recent efforts,^54–58^ the Schäfer group reported the successful synthesis of main-chain cobaltocenium-containing polymers with only one methylene group as a linker.^59^ This design yields a main-chain polymer, polycobaltoceniumylmethylene, with a high cationic charge density per polymer chain. Ring-opening transmetalation polymerization (ROTP) was performed using Me_2_C­[1]­magnesocenophane and cobalt­(II) chloride, then subsequent oxidation with ammonium chloride to synthesize the main-chain cobaltocenium polymer (**PCo-MC**) illustrated in Scheme 1.^59^ The polymer was characterized with ^1^H NMR, which has a molecular weight of approximately 16.3 kDa (Figures S1, S2). According to our early work, side-chain cobaltocenium polymer (**PCo-SC**) was prepared by reversible-addition–fragmentation transfer (RAFT) polymerization using cobaltocenium aminoethyl methacrylate hexafluorophosphate as a monomer, 2-cyano-2-propyl benzodithioate (CPB) as chain transfer agent, and 2,2’-azobis­(isobutyronitrile) (AIBN) as an initiator illustrated in Scheme 1.^50^ ^1^H NMR confirmed the successful polymerization of **PCo-SC**, with a molecular weight of approximately 22.7 kDa (Figures S1, S2).

**Scheme 1:**
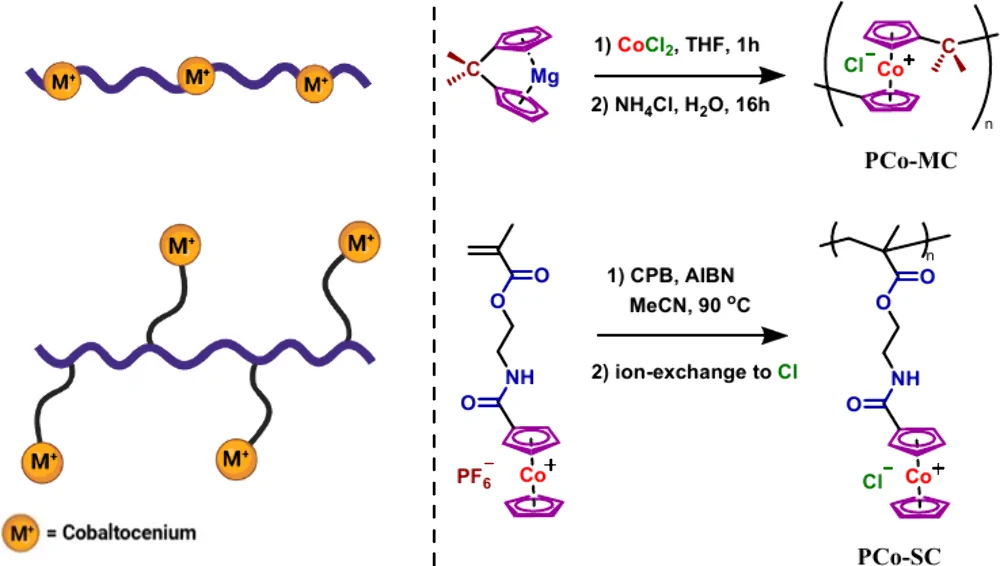
Synthesis of Cobaltocenium-Containing Main-Chain Polymer (**PCo-MC**) and Side-Chain Polymer (**PCo-SC**)

Previous studies have established that the optimal molecular weight for an effective antimicrobial polymer or polypeptide is typically in the range of 5.0–30.0 kDa.^50,60,61^ Thus, we chose the above-mentioned molecular weight in a similar range for the current proof-of-concept study. It is worth mentioning that the exact compositions between main-chain and side-chain polymers cannot be achieved at this stage due to the lack of available synthetic methodologies.

### Antimicrobial Activity and Selectivity of Main-Chain and Side-Chain Polymers

To compare the antimicrobial activity of main-chain vs side-chain polymers, we first assessed the minimum inhibitory concentration, MIC of both polymers against different laboratory strains (Table 1). The main-chain polymer has much higher antimicrobial activity compared to its counterpart, with an MIC value of 8–16 μg/mL, whereas the side-chain polymer was essentially non-antimicrobial (MIC ≥ 256 μg/mL). When tested against methicillin-resistant *S. aureus* (MRSA) and clinical isolates of Gram-negative bacteria, it reflected a similar trend of activity (Table 1). In the case of Gram-positive MRSA strains, MIC was lowest for the main-chain polymer (4 μg/mL), 16–32 fold lower compared to the side-chain polymer. For Gram-negative bacterial strains, **PCo-MC** was significantly effective at inhibiting bacterial growth, while **PCo-SC** resulted in no significant antimicrobial effect.

**Table 1:** Antimicrobial Activity and Selectivity of Cobaltocenium-Containing Polymers^a^

	MIC (μg/mL)		MBC of PCo-MC (μg/mL)		Selectivity (HC_50_/MIC)	
Bacteria strain	PCo-MC	PCo-SC	Planktonic cells	Stationary cells	PCo-MC	PCo-SC
*A. baumannii* (ATCC-19606)	8	256	ND^a^	ND^a^	>128	>4
*E. cloacae* (ATCC-13047)	8	>256	ND^a^	ND^a^	>128	>4
*P. aeruginosa* (ATCC-10145)	16	>512	ND^a^	ND^a^	>64	>2
*E. coli* (ATCC-11775)	8	512	ND^a^	ND^a^	>128	>2
MRSA (BAA-1717)	4	128	4	4	>256	>8
MRSA (BAA-44)	4	64	4	4	>256	>16
MDR *A. baumannii* (BAA-1797)	8	512	8	16	>128	>2
MDR *E. hormaechei* (BAA-2468)	8	64	8	8	>128	>16
MDR *E. coli* (BAA-197)	8	512	8	8	>128	>2
MDR *E. coli* (BAA-2452)	8	512	8	8	>128	>2
MDR *E. coli* (BAA-2471)	8	512	8	8	>128	>2
MDR *P. aeruginosa* (BAA-2110)	16	>256	16	8	>128	<4
MDR *P. aeruginosa* (BAA-2108)	16	512	16	32	>64	>2
MDR *K. pneumoniae* (BAA-2473)	8	>256	8	8	>128	<4

a ND, not determined.

b Minimum inhibitory concentration (MIC) was determined according to the CLSI protocol.^60^ Minimum bactericidal concentration (MBC) was assessed for MDR bacteria strains of both planktonic phase and stationary phase. Selectivity was based on hemolytic activity of both polymers.

The stark contrast of **PCo-MC** vs **PCo-SC** can be attributed to the alternating sequence of cationic charge and hydrophobicity in the main-chain polymer, which resulted in enhanced interaction with negatively charged bacterial membrane and facilitated the insertion of hydrophobic segment into the lipophilic portion of the membrane.^62^ However, both the main-chain and side-chain polymers exhibited greater activity against Gram-positive strains compared to Gram-negative strains. This difference in activity can be explained by cell-wall architecture, as Gram-positive bacteria only possess a single, thick peptidoglycan layer, whereas Gram-negative strains have an additional outer membrane rich in lipopolysaccharides. Such a membrane barrier in Gram-negative bacteria reduces their susceptibility to larger hydrophobic drugs as well as AMP-mimic polymers.^63^

The efficient antimicrobial effect of **PCo-MC** prompted us to evaluate if the polymer is bactericidal or bacteriostatic in nature. Based on differences in growth conditions, bacteria can grow into two subpopulations: planktonic phase and stationary phase. Planktonic phase bacteria have all the basic metabolisms operating under normal condition, but stationary phase bacteria alter their metabolic activities to conserve energy and render many antibiotics ineffective.^8^ To measure the minimum bactericidal concentration (MBC), we used both planktonic and stationary phase bacteria of MDR bacterial strains. The MBC values ranged from 8 to 32 μg/mL (Table 1), indicating a bactericidal effect at relatively lower concentrations for both phases.

Biocompatibility is a crucial parameter that can assess the applicability of an antimicrobial agent. To evaluate biocompatibility, we need to confirm that a polymer is selective to bacterial cells and not harmful for mammalian cells.^12^ Selectivity is a therapeutic index that reflects the applicability of an antimicrobial polymer. Both polymers were exposed to mouse red blood cells (RBCs) to determine the toxicity and were found to be nontoxic at the maximum test concentration of 1024 μg/mL. Based on the value, the selectivity was reported as HC_50_/MIC, where HC_50_ is the concentration required to cause 50% lysis of RBCs (Table 1). Although both polymers are nontoxic in nature, the selectivity of **PCo-MC** is much higher than **PCo-SC** due to the lower MIC values for the former.

### Killing Kinetics and Pairwise Statistical Analysis

Rapid bacterial eradication, which minimizes the likelihood of resistance development, is essential for designing effective AMP-mimicking polymers. **PCo-MC** was evaluated for killing kinetics against two MDR bacterial strains, while imipenem was used as a control antibiotic. For MDR *E. coli* (BAA-2452), the polymer exhibited a dose-dependent killing efficiency with 4× MIC and 2× MIC eradicating the whole bacterial population (∼6-log reduction) within 40 min, while at the MIC it took 60 min to achieve complete killing. Sub-MIC, positive control, and imipenem showed no significant reduction of bacterial load until 12 h (Figure 2A). MDR *K. pneumoniae* (BAA-2473) showed a similar bactericidal effect upon polymer treatment with a steep decline of bacterial population with 4× MIC and 2× MIC within 20 min, while at MIC, it exhibited complete elimination at 90 min (Figure 2B). Against both strains at 20 min, 4× MIC and 2× MIC exhibited 3-log reduction compared to the control, indicating a swift killing rate. Additionally, sub-MIC, control, and imipenem again showed no killing effect in growth.

**Figure 2:**
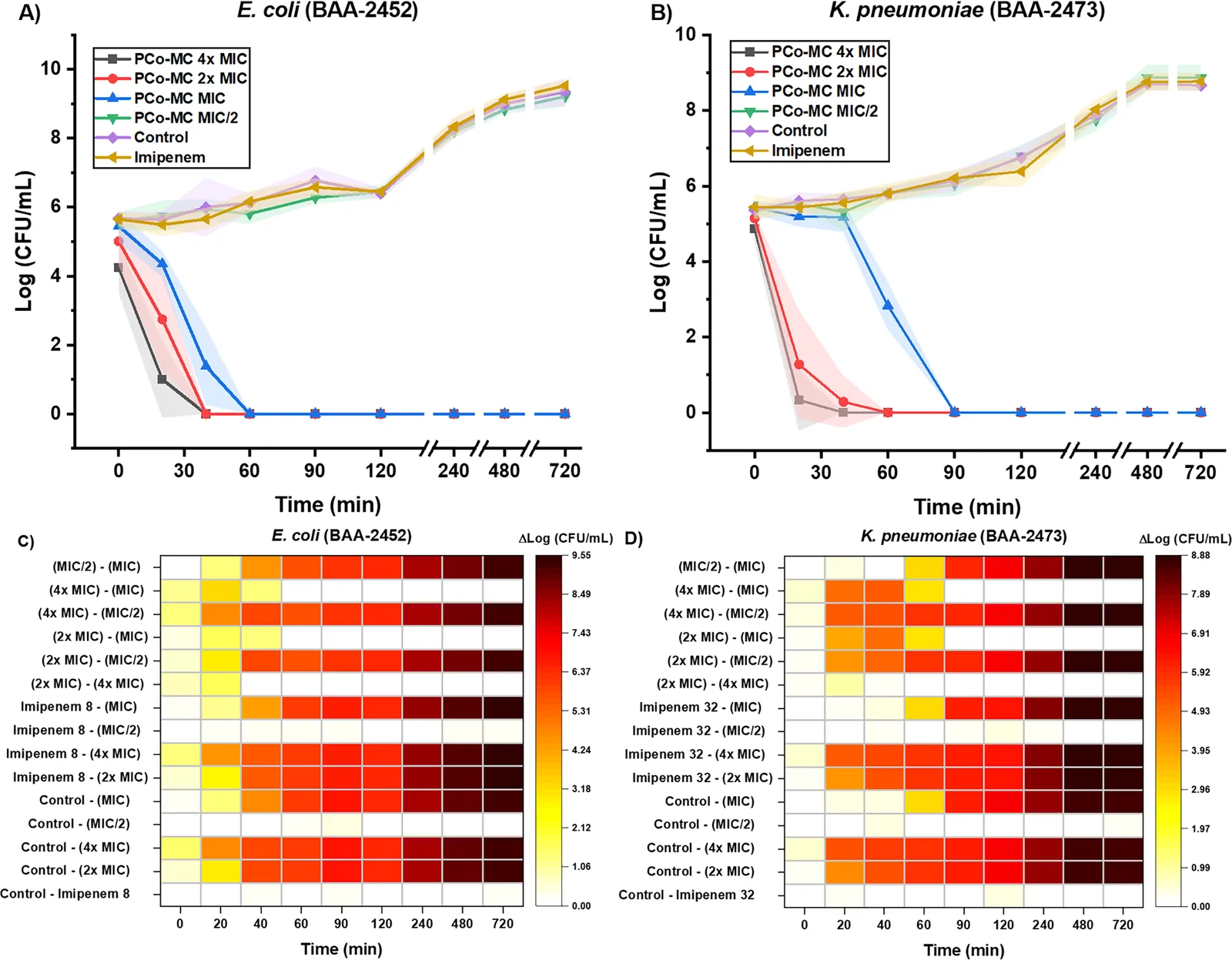
Time-dependent killing kinetics of **PCo-MC** against two MDR bacteria (MIC of PCo-MC against both strain is 8 μg/mL): A) *E. coli* (BAA-2452); B) *K. pneumoniae* (BAA-2473). Pairwise comparison of six treatment groups for each time points: control, **PCo-MC** (MIC, MIC/2, 2× MIC, 4× MIC), and antibiotic (Imipenem 8 μg/mL for *E. coli* and 32 μg/mL for *K. pneumoniae*); C) *E. coli* (BAA-2452); D) *K. pneumoniae* (BAA-2473).

Further, we performed pairwise comparison of six treatment groups against both bacterial strains for the killing kinetics to identify pairs of treatment groups for which a significant difference was observed. The heatmap in Figure 2C and D represents time of data acquisition and the pair of treatment groups as independent variables, and the estimated difference of bacterial load, ΔLog (CFU/mL) as the dependent variable. For both bacteria, there is no significant difference in bacterial load that appears at the initial time point, *t* = 0 min. At *t* = 20 min, three pairs showed obvious contrast consisting of 4× MIC versus control, imipenem, and sub-MIC for *E. coli* (Figure 2C). For *K. pneumoniae* (Figure 2D), a group of eight pairs including 4× MIC, 2× MIC versus control, imipenem, MIC and sub-MIC exhibited similar significant differences. After *t* = 60 min, all three treatments (MIC, 2× MIC, and 4× MIC) showed significant reduction of bacterial growth when paired with control, imipenem, and sub-MIC across both bacterial strains. In Table 2, we highlight some of the treatment pairs and show the change in *p*-values at different times. Pairwise analysis for each time point for all treatment groups is included in the Supporting Information. No significant difference was observed within pairs of control, imipenem, and sub-MIC throughout the experimental time points.

**Table 2:** Pairwise Comparison of Treatment Groups with Key Pairs up to 60 Min of Treatment^a^

	MDR *E. coli* (BAA-2452)			MDR *K. pneumoniae* (BAA-2473)		
Time	Treatment group (*p*-value)			Treatment group (*p*-value)		
0 min	Control – 2× MIC (0.0505)	Control – 4× MIC (<0.0001)	2× MIC – 4× MIC (0.0131)	Imipenem – 4× MIC (0.0317)	4× MIC – MIC (0.0419)	2× MIC – 4× MIC (0.6796)
20 min	Control – MIC (<0.0001)	2× MIC – MIC (<0.0001)	2× MIC – 4× MIC (<0.0001)	Imipenem – 2× MIC (<0.0001)	2× MIC – MIC (<0.0001)	2× MIC – 4× MIC (<0.0001)
40 min	Imipenem – MIC (<0.0001)	Control – Imipenem (0.6851)	MIC/2 – MIC (<0.0001)	Imipenem – MIC (0.3595)	Control – Imipenem (0.9941)	Control – 2× MIC (<0.0001)
60 min	Imipenem – MIC (<0.0001)	Control – Imipenem (1.0000)	2× MIC – MIC (<0.0001)	Imipenem – MIC (<0.0001)	Control – Imipenem (1.0000)	2× MIC – MIC (<0.0001)

a Pairs with significant differences have *p*-value < 0.05.

### Anti-Biofilm Activity

Biofilms are a form of bacterial resistance, where an opportunistic pathogen can adhere to either abiotic or biotic surfaces and form a protective layer of extracellular polymeric substances (EPSs) to prevent them from antimicrobial agents. Biofilms consist of persister cells, which can escape most of the antibiotics due to change in metabolic activities.^64,65^ Therefore, we investigated the efficacy of our polymer to the mature biofilm of two representative Gram-negative clinical isolates, also known to cause nosocomial infections: MDR *P. aeruginosa* (BAA-2110, labeled as Pa-1) and MDR *P. aeruginosa* (BAA-2108, labeled as Pa-2). First, biofilms with and without treatment were stained with crystal violet to assess the reduction of biomass formation. From Figure 3A and B, it is evident that **PCo-MC** diminished the biomass by 70–80% and 55–65% respectively against Pa-1 and Pa-2 at different dosages (4× MIC to 16× MIC). In contrast, polymyxin-B was required in a higher dose (32× MIC) compared to **PCo-MC** to exhibit likewise efficacy. A Gram-positive MRSA strain (BAA-1717) was also evaluated for biofilm treatment (Figure 3C). The polymer achieved approximately 30% biomass reduction at 2× MIC, whereas vancomycin, a cell-wall synthesis inhibitor, reduced the original biomass by 50–55%.

**Figure 3:**
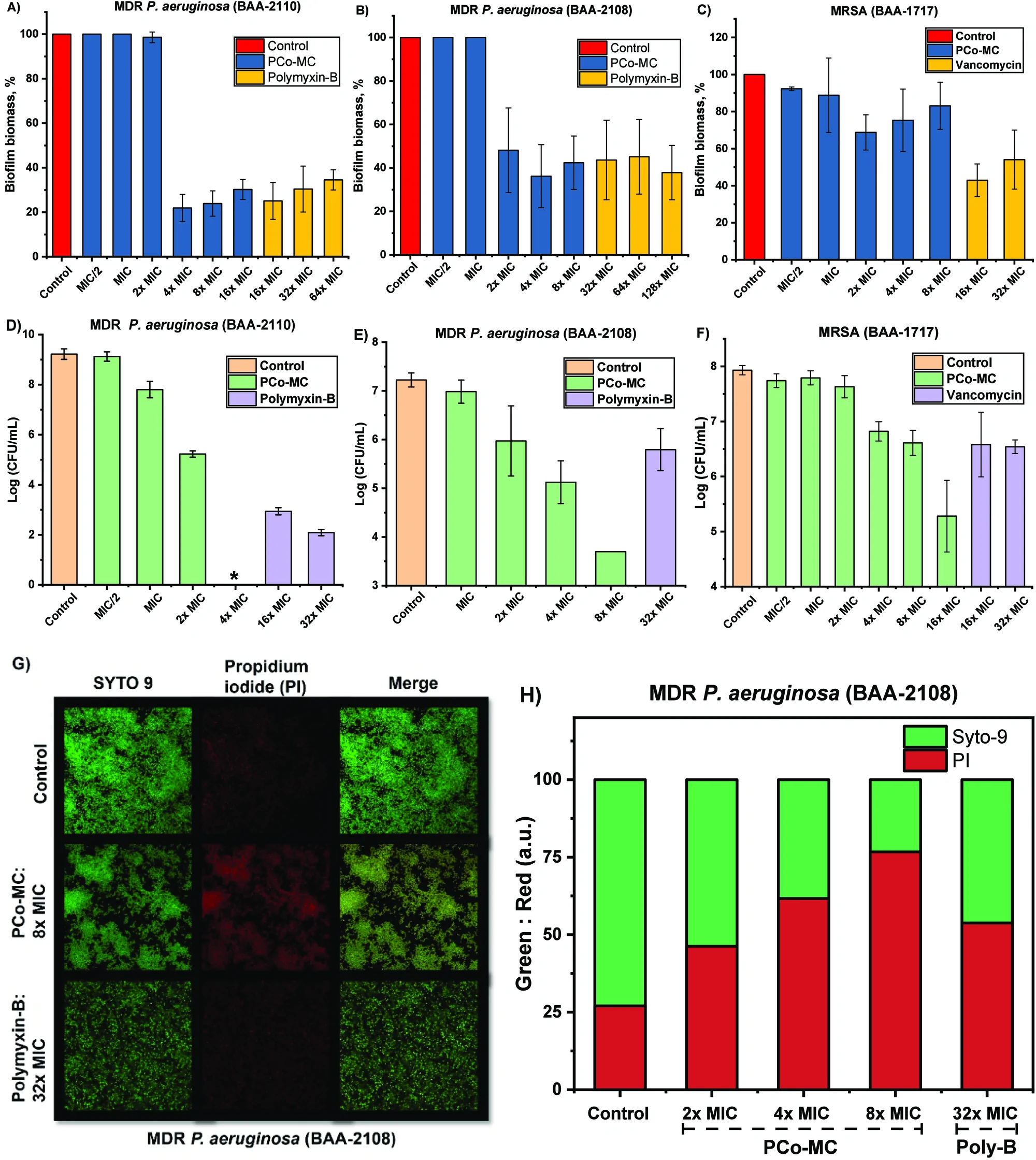
Treatment of mature biofilms of Gram-negative and Gram-positive bacteria by using **PCo-MC**. Biofilm biomass assay: A) MDR *P. aeruginosa* (BAA-2110), B) MDR *P. aeruginosa* (BAA-2108), and C) MRSA (BAA-1717); biofilm-embedded cell viability: D) MDR *P. aeruginosa* (BAA-2110), E) MDR *P. aeruginosa* (BAA-2108), and F) MRSA (BAA-1717); G) confocal fluorescence microscopy images of live/dead MDR *P. aeruginosa* (BAA-2108) bacterial cells; H) mean area coverage of green/red stained MDR *P. aeruginosa* (BAA-2108) bacterial cells after treatment.

Biomass measurements reflect the extent of biofilm disruption but do not quantitatively capture the killing of biofilm-embedded bacteria. Therefore, we assessed the cell-viability of biofilm embedded bacteria, which contain dormant cells including persister. For Pa-1, a complete eradication of bacterial cells was achieved at 4× MIC. Surprisingly, polymyxin-B at even 32× MIC failed to eradicate them completely (Figure 3D). In the case of Pa-2, 1-log reduction (90% killing) was observed at 32× MIC of polymyxin-B, whereas **PCo-MC** achieved a 3.5-log reduction at 8× MIC (Figure 3E). Additionally for MRSA biofilm, a 2.5-log reduction occurred at 16× MIC of **PCo-MC**, compared to 1-log reduction by 16× and 32× MIC of vancomycin (Figure 3F). These results illustrated high efficacy of the main-chain polymer compared to the best antibiotic treatments currently offered.

To strengthen the anti-biofilm activity of **PCo-MC**, we further performed a live/dead assay of biofilm-embedded bacteria using a dye mixture of SYTO 9 (green emission, nucleic acid stain) and propidium iodide (PI, red emission, nucleic acid stain) through fluorescence imaging. SYTO 9 is a cell permeable stain, which shows both live and dead cells; in contrast PI is cell impermeable and can only stain dead cells. From Figure 3G, it was evident that **PCo-MC** at 8× MIC showed a better elimination of bacterial cells, compared to 32× MIC of polymyxin-B. In addition, we calculated the mean area coverage by the dye mixture and calculated the percentage of green and red areas shown in Figure 3H, which further corroborates our findings from the cell viability assay. Overall, these results demonstrated that **PCo-MC** exhibited potent and consistent anti-biofilm activity, effectively disrupting biofilm structure and eradicating embedded bacterial cells, outperforming conventional antibiotics in both efficacy and dose efficiency.

### Mechanisms of Action

After assessing the antimicrobial efficacy and anti-biofilm activities, one needs to understand the mechanisms of action at both molecular and cellular levels. We carried out the investigation using a holistic and comprehensive approach with several aspects of polymer–cell interactions.

#### Membrane Depolarization and Permeabilization

To assess the cytoplasmic depolarization effect, a membrane-potential sensitive dye 3,3′-dipropylthiadicarbocyanine iodide, DiSC_3_ (5) was used in combination with **PCo-MC**.^50,52,60^ In Figure 4A, the polymer was used as treatment against MDR *K. pneumoniae* (BAA-2473) at varying concentrations- 2× MIC, 4× MIC, and 8× MIC. A dose-dependent increase in fluorescence was observed as the treatment concentration increased, indicating significant membrane depolarization. Cationic polymers are believed to perturb the outer membrane through electrostatic and lipophilic interactions. To gain mechanistic insights of **PCo-MC**, we used 1-*N*-phenylnaphthylamine (NPN) dye, which indicates outer membrane permeabilization.^50,52,60^ In Figure 4B, polymer treatment showed an increase in fluorescence of NPN dye upon addition to MDR *K. pneumoniae* (BAA-2473), indicating damage to the outer membrane. Inner membrane permeabilization by **PCo-MC** was investigated by treating *E. cloacae* (ATCC-13047) bacteria containing PI dye and recording fluorescence.^66^ The fluorescence intensity gradually increased with all **PCo-MC** concentrations illustrating damage to the inner membrane of bacteria (Figure 4C).

**Figure 4:**
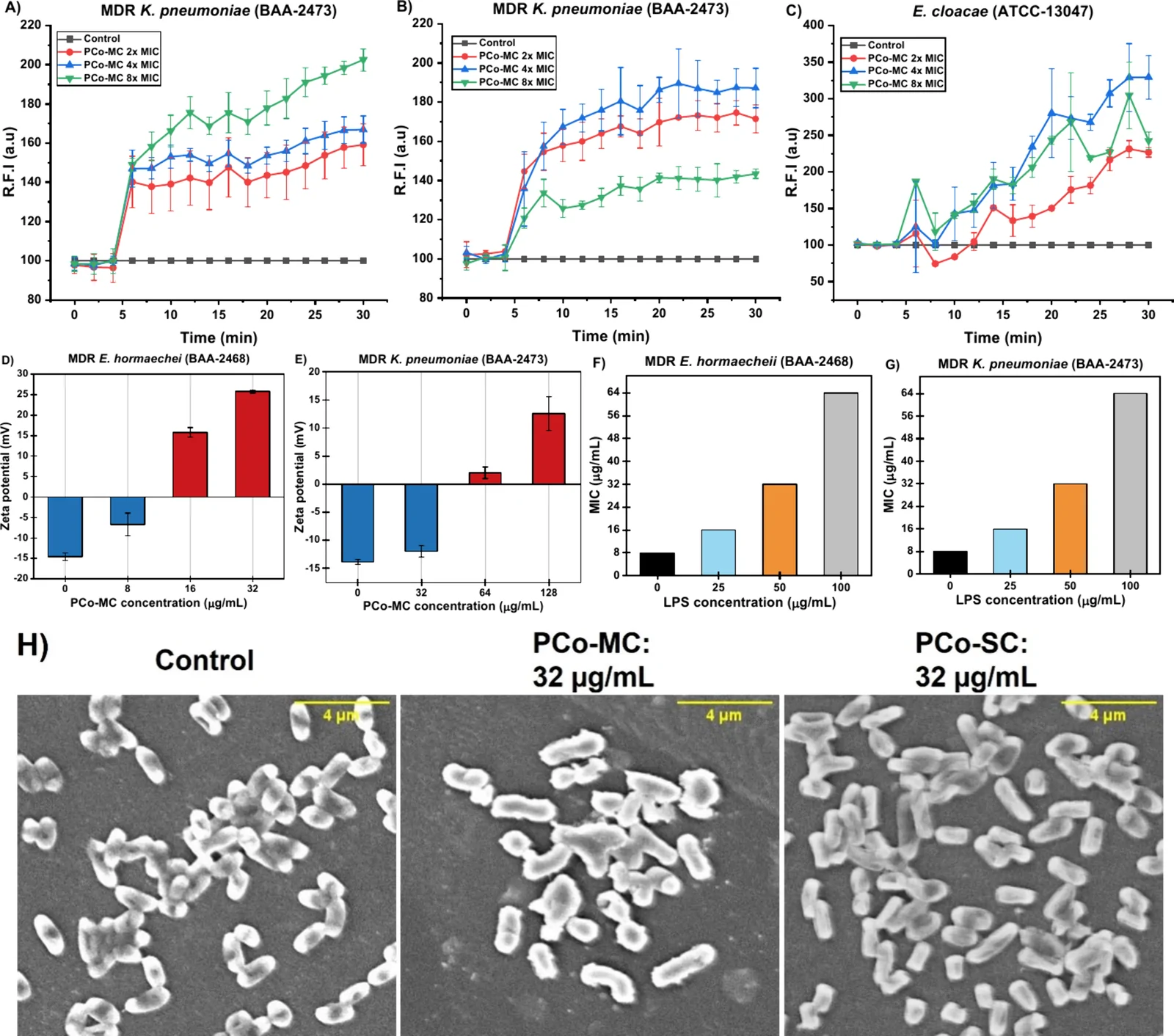
A) Cytoplasmic membrane depolarization of MDR *K. pneumoniae* (BAA-2473); B) outer membrane permeabilization of MDR *K. pneumoniae* (BAA-2473); C) inner membrane permeabilization of *E. cloacae* (ATCC-13047); surface charge alteration of bacterial cells: D) MDR *E. hormaechei* (BAA-2468) and E) MDR *K. pneumoniae* (BAA-2473); lipopolysaccharide inhibition assay: F) MDR *E. hormaechei* (BAA-2468) and G) MDR *K. pneumoniae* (BAA-2473); H) SEM images of MDR *K. pneumoniae* (BAA-2473).

The electrostatic interaction between polymer and bacteria can be measured by taking zeta potential measurements. For MDR *E. hormaechei*, the initial zeta potential was −14.53 mV. After adding only 8 μg/mL of **PCo-MC** the potential shifted to −6.67 mV, while at 16 μg/mL the zeta-potential became +15.8 mV, and at 32 μg/mL the potential was +25.73 mV (Figure 4D). Additionally, MDR *K. pneumoniae*, also exhibited charge alteration with an initial voltage of −13.87 mV shifting to a final zeta potential of +12.57 mV upon adding 128 μg/mL of **PCo-MC** (Figure 4E). These findings exhibited strong electrostatic interaction between the polymer and bacterial cells by alteration of surface charge in the bacterial population.

#### Lipopolysaccharide (LPS) Inhibition and Bacterial Cell Morphology

LPS molecules are an integral part of the outer membrane, which renders the membrane negatively charged. **PCo-MC** was used to assess the MIC of different Gram-negative bacteria in the presence of varying concentrations of LPS. MIC of the polymer against both bacterial strains is 8 μg/mL. With increasing concentrations of externally added LPS, the MIC value kept increasing against both MDR *E. hormaechei* and MDR *K. pneumoniae* (Figure 4F,G). These results indicate that the polymer strongly interacts with LPS molecules present in bacteria. Together with the membrane depolarization and permeabilization data, this supports a membrane-active mechanism of action.

The effect of the cobaltocenium polymer on bacterial cells was visualized through scanning electron microscopy (SEM). In Figure 4H, untreated bacteria can be seen with a healthy rod-shaped morphology. After treatment with **PCo-MC**, the bacteria were deformed. However, in the case of **PCo-SC**, it was observed that bacteria maintain their smooth and regular rod-shaped morphology, which indicated that this polymer cannot exert a detrimental effect on the cell. These results suggest that the main chain polymer likely acts as a membrane-active agent, inducing substantial damage to the bacterial cell envelope.

### Inhibition of Antimicrobial Resistance

Resistance development was assessed against MDR bacteria toward the main-chain polymer. Two MDR strains were tested separately to evaluate the efficacy of **PCo-MC** for 22 days of repeated treatment. Polymyxin-B was used as an antibiotic control. As shown in Figure 5A, the polymer did not become resistant at all, whereas polymyxin-B became ineffective on the second day of treatment, reaching as high as 8192-fold of its initial MIC after 20 days (MIC of polymyxin-B against MDR *K. pneumoniae* (BAA-2473) was 0.5 μg/mL). For MDR *E. hormaechei* (BAA-2468) in Figure 5B, a similar phenomenon was noticed, as **PCo-MC** remains active at 8 μg/mL throughout the experiment (MIC of polymyxin-B against MDR *E. hormaechei* (BAA-2468) was 0.25 μg/mL). This demonstrates the superior efficacy of **PCo-MC** over conventional antibiotics with no detectable resistance developed.

**Figure 5:**
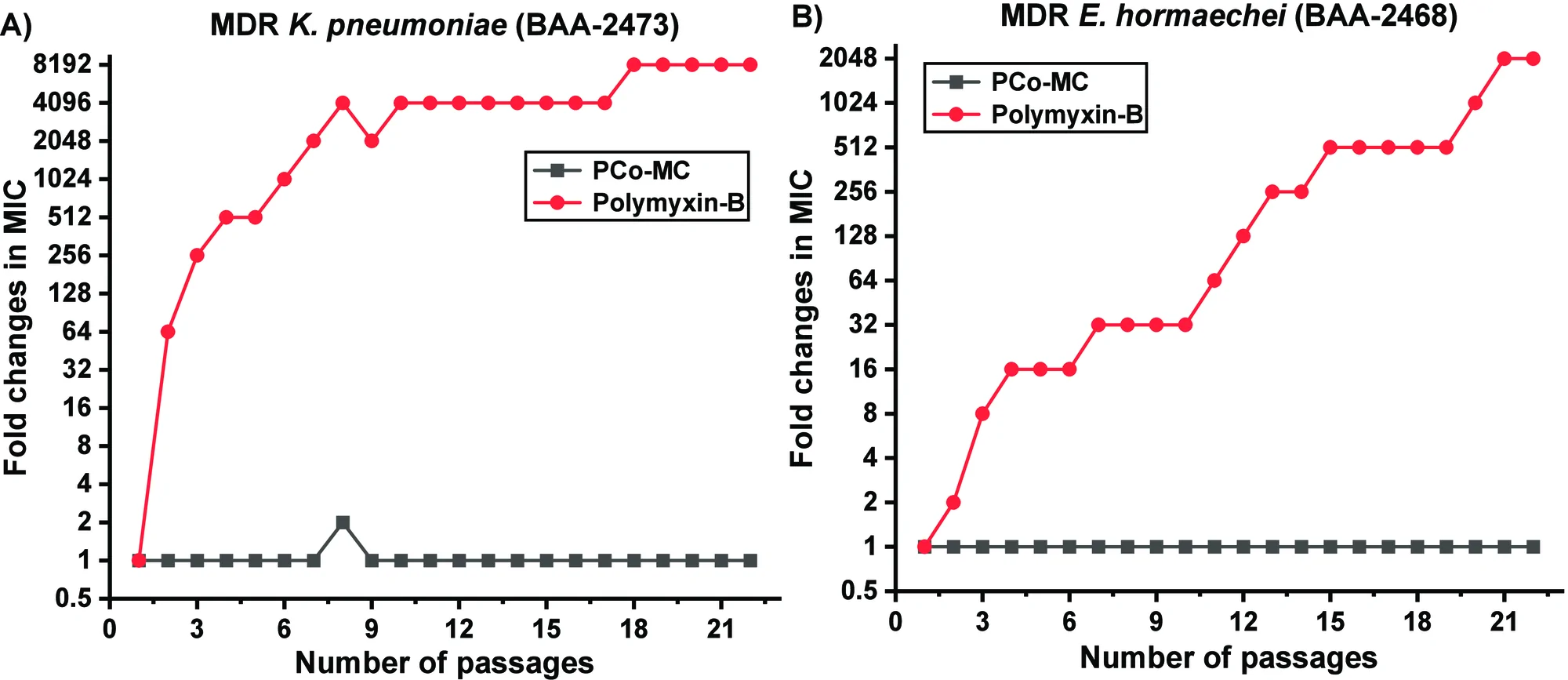
Resistance development assay of **PCo-MC** against Gram-negative bacteria: A) MDR *K. pneumoniae* (BAA-2473); B) MDR *E. hormaechei* (BAA-2468).

## Conclusions

Spatial control plays a critical role in the design of antimicrobial polymers. In this study, a main-chain cobaltocenium polymer was evaluated as an antimicrobial agent and, in comparison to its side-chain analogue, exhibited highly selective and broad-spectrum activity against both planktonic and stationary-phase bacteria. Time–kill kinetics demonstrated rapid, dose-dependent bacterial eradication, supported by statistically rigorous kinetic analyses and pairwise comparisons of treatment groups.

The active polymer further displayed significant anti-biofilm activity against both Gram-negative and Gram-positive bacterial strains. Mechanistic investigations suggested a membrane-active mode of action, as evidenced by outer- and inner-membrane permeabilization, membrane depolarization, LPS interaction, and alterations in bacterial surface charge. Finally, resistance studies conducted over 22 days against MDR Gram-negative bacterial strains revealed no detectable resistance development in contrast to conventional antibiotics.

Overall, these findings highlight metallopolymers as a promising alternative antimicrobial platform and underscore the importance of spatial control of cationic centers as a key design parameter for enhancing the efficacy of the antimicrobial polymers.

## Supplementary Material



## Notes

Details on experimental methods, measurement of biological activity and characterization; ^1^H NMR, GPC chromatograms, and hemolysis assay of cobaltocenium polymers; pairwise comparison analysis at different time points are included for two bacterial strains (PDF)
