# Supplementary material for: Spatial Evolution of Antimicrobial Metallopolymers toward the Main Chain Topology
Source: ACS Cent Sci. 2026 Jun 19;12(8):1099–109. doi: 10.1021/acscentsci.6c00694 (PMC13523504; doi:10.1021/acscentsci.6c00694)
Supplement: Supplementary file 1 [file oc6c00694_si_001.pdf]

Supporting Information

Spatial Evolution of Antimicrobial Metallopolymers Toward the Main Chain Topology

Md Waliullah Hossain,[a] Marsh Kral,[a] Aylin Feuerstein,[b] Swagatam Barman,[a] Alimi Abiodun,[a] Markus Gallei,[b,c] Chao Cai,[d] André Schäfer,[b] and Chuanbing Tang[a*]

[a] Department of Chemistry and Biochemistry, University of South Carolina, Columbia, South Carolina 29208, USA
[b] Department of Chemistry, Faculty of Natural Sciences and Technology, Saarland University, 66123 Saarbrücken, Germany
[c] Saarland Center for Energy Materials and Sustainability – Saarene, Saarland University, 66123 Saarbrücken, Germany
[d] Clinical Pharmacy and Outcomes Sciences (CPOS), College of Pharmacy, University of South Carolina, Columbia, South Carolina 29208, USA

[*] Corresponding Author Email: tang4@mailbox.sc.edu

Table of Contents

Experimental methods and materials (Pages S2-S7)

Supporting Figures and Table (Pages S7-S20)

Figure S1: ^{1}H NMR spectra of polymers

Figure S2: GPC traces of polymers

Figure S3: Hemolysis of mouse red blood cells (RBCs) by polymers

Pairwise comparison analysis for each time points of killing kinetics

References (Pages S20)

Experimental Methods and Materials

Materials: All reagents were used as received unless otherwise stated. Antibiotics were purchased from Chem-Impex Chemicals. Dyes for biological assays were bought from Fisher Scientific. 10× PBS was purchased from MP Biomedicals. Deuterated solvent, deuterium oxide D_2O and dimethyl sulfoxide DMSO-d_6, were purchased from Cambridge Isotope Laboratories. All bacterial strains, including multidrug-resistant (MDR) strains, were purchased from ATCC, USA. The general supplies for microbiological studies (e.g., bacterial media, buffers, well plates, centrifuge tubes etc.) were brought from VWR International.

Instrumentation: Bruker Avance IV HD 400 spectrometer was employed to record proton Nuclear Magnetic Resonance (NMR) spectrum of monomers and polymers. In general, samples (5-8 mg) were dissolved in appropriate deuterated NMR solvents of ~600 µL before the NMR experiments. Zeta potential measurements of the polymers were conducted using a Zetasizer Nano ZS (Malvern Instruments, Malvern, UK), equipped with a 4.0 mW 633 nm He-Ne laser and a detector positioned at 173 degrees. Data analysis was conducted using the general-purpose algorithms available in Zetasizer Software (version 7.11). Each sample measurement was carried out in triplicate, and results are presented as the average at a temperature of 25 °C. Each sample was prepared by dissolving the dry polymer in filtered deionized water. The typical concentration for all samples was 10.0 mg/mL.. Optical density (OD) and fluorescence intensity measurements for the biological assays were conducted using a SpectraMax M5 Multimode Microplate Reader (Molecular Devices). Zeiss Gemini500 FE-SEM was employed to perform electron microscopy of the bacteria. Zeiss LSM 700 Confocal Laser Scanning Microscope in 63× magnification was used for bacterial biofilm live/dead fluorescence assay. Deionized water was purified using a Millipore water purification system with a minimum resistivity of 18.2 megaohms/cm.

Synthesis of side-chain cobaltocenium polymer (PCo-SC): Cobaltocenium-containing methacrylate polymers with hexafluorophosphate anion and its chloride paired cobaltocenium-containing polymers were synthesized according to the earlier reports.(1, 2) Cobaltocenium-2-aminoethyl methacrylate (CoAEMA) was polymerized by using the Reversible Addition–Fragmentation Chain Transfer (RAFT) polymerization technique. A typical protocol was as follows. In a 25 mL Schlenk flask the monomer CoAEMA (1 g, 2.04 mmol) was first dissolved in anhydrous acetonitrile (1.5 mL) before adding AIBN (1.5 mg, 0.006 mmol) and CPB (10.03 mg, 0.03 mmol). A stock solution of both AIBN and CPB was prepared for all polymerizations. After three freeze–pump–thaw cycles, the flask was filled with nitrogen. The flask was immersed in a preheated oil-bath set at 90 °C for 5 h. The reaction mixture was quenched by cooling it in an ice bath and exposing it to air. The polymer solution was precipitated out in excess dichloromethane, and the solid polymer was collected. After drying, it was redissolved in acetonitrile and precipitated at least 3 times. Later, the yellow polymer was dried under vacuum to obtain as final product. The side-chain polymer (PCo-SC) was confirmed by ^{1}H NMR (400 MHz, DMSO-d6): δ = 0.50–1.01 (3H, –CH_2-C-C$\boldsymbol{H_3}$), δ = 1.48–2.03 (2H, –C$\boldsymbol{H_2}$-C-CH_3), δ = 3.38–3.62 (2H, –CH_2-

C$\boldsymbol{H_2}$-NH-), δ = 3.84–4.33 (2H, –O-C$\boldsymbol{H_2}$-CH_2), δ = 6.28–6.75 (2H, Cp), δ = 5.72–6.19 (7H, Cp) δ = 7.08–7.84 (5H, phenyl).

Synthesis of main-chain cobaltocenium polymer (PCo-MC): Cyclopentadiene was obtained from dicyclopentadiene by cracking and distillation. Me2C[1]magnesocenophane was synthesized according to literature-known procedures.(3-5) Under an atmosphere of argon, Me2C[1]magnesocenophane (1.50 g, 7.70 mmol, 1.0 eq) and cobalt(II) chloride (1.00 g, 7.70 mmol, 1.0 eq) were charged into a Schlenk flask and 100 ml THF were added. The resulting reaction mixture was stirred for 1 h at room temperature. Afterwards, 100 ml of an aqueous ammonium chloride (824 mg, 15.4 mmol, 2.0 eq) solution were added and the mixture was stirred for additional 16 h under ambient atmosphere. The solvents were removed *in vacuo*, and a green colored solid was obtained. The solid was suspended in methanol and added dropwise into vigorously stirring THF, to obtain a green precipitate. The solid was collected and heated to 130 °C *in vacuo* to remove excess NH_4Cl. Afterwards, 120 ml of water was added, and the water-insoluble solid was filtered. The yellow-brownish solution was transferred into a dialysis tube (MWCO: 3.5 kD), and dialysis was performed against deionized water, while a yellow coloring of the medium outside the dialysis tube was observed at the beginning (water was renewed 18 times). After 48 h no coloring of the medium outside the dialysis tube was observed. The clear brownish solution was evaporated to dryness and a dark green-blackish solid was obtained. The obtained dark green solid was dried *in vacuo* at 60 °C. Synthesis of main-chain cobaltocenium polymer (PCo-MC) was confirmed by ^{1}H NMR (400 MHz, D_2O): 5.86 (bs, 4H, Cp-H); 5.82 (bs, 4H, Cp-H); 1.78 (s, 6H, CH3).

Measurement of antibacterial activity

Escherichia coli (ATCC-11775), *Pseudomonas aeruginosa* (ATCC-10145), *Enterobacter cloacae* (ATCC-13047), *Acinetobacter baumannii* (ATCC-19606), MRSA-1 (BAA-1717), MRSA-2 (BAA-44), MDR *A. baumannii* (BAA-1797), MDR *E. coli*-1 (BAA-197), MDR *E. coli*-2 (BAA-2452), MDR *E. coli*-3 (BAA-2473), MDR *E. hormaechei* (BAA-2468), MDR *K. pneumoniae* (BAA-2473), MDR *P. aeruginosa*-1 (BAA-2108), and MDR *P. aeruginosa*-2 (BAA-2110) were purchased from ATCC. Bacteria were streaked on tryptic soy agar (TSA) plates from their primary glycerol stock. After incubation at 37 °C for 24 h, a single colony was inoculated in 3 mL of tryptic soy broth (TSB) at 37 °C for 6 h under constant shaking at 190 rpm to reach the mid-log phase. Specific antibiotics were used in both TSA plates and TSB for specific bacteria: imipenem (25 μg mL^{-1}) for MDR *E. coli*-2 (BAA-2471), MDR *E. cloacae* (BAA-2468), and MDR *K. pneumoniae* (BAA-2473). All bacteria were adjusted to an optical density of 0.5 McFarland standard (OD_{600} = 0.70) for further use.

Minimum inhibitory concentration (MIC) assay: The MIC assay was performed with slight adjustment to the reported protocol.(6) Different concentrations of polymers in 100 μL of normal saline solution using a 2-fold dilution method were added to 96-well plates. Then, 100 μL of bacteria containing CAMHB solution (~10^5 CFU per mL) was transferred to each well. Bacterial solutions and media without compounds were used as the positive control, and only media with compounds were used as the negative control. The prepared 96-well plate was placed in an incubator at 37 °C and shook at 190 rpm for 16–18 h. The bacterial growth was measured by

reading OD_{600} values. The MIC_{90} was determined as the lowest concentration of the compound that completely inhibited at least 90% of bacterial growth. Each test was performed three different times, with triplicate measurements for accuracy.

Minimum Bactericidal Concentration (MBC) Assay: Mid-log phase (~10^8 CFU/mL) bacterial cell cultures were suspended to ~10^5 CFU/mL in media. 150 µL of this bacterial suspension was mixed with 50 µL of MIC/2, MIC, 2× MIC, 4× MIC, 8× MIC of the corresponding compound followed by incubation at 37 °C overnight. As a negative control, the same volume (50 µL) of water was used instead of any antibacterial compounds. Samples of 20 µL of each concentration were then 10-fold serially diluted in 1× PBS. From these dilutions, 20 µL was spot plated on a tryptic soy agar plate and incubated for 24 h at 37 °C for counting viable cells. These experiments were performed in triplicate.

Hemolytic activity: All experimental procedures were approved by the University of South Carolina Institutional Animal Care and Use Committee (IACUC) under AUP# 2508, following the guidelines of the National Research Council for the Care and Use of Laboratory Animals. Blood was collected from mice in heparinized tubes and washed with PBS containing 10% FBS. Polymers were prepared in PBS at varying concentrations and added to the blood in a 96-well plate (150 µL per well). PBS with 0.2% Triton X-100 served as the maximal lysis control. The samples were incubated at 37 °C for 1 hour and then centrifuged at 400g for 10 minutes. Following centrifugation, 100 µL of supernatant from each well was transferred to a new 96-well plate, and absorbance was measured at 450 nm. PBS with 10% FBS was used for background readings. The hemolysis was calculated as: $\frac{Sample\ OD - background}{Max\ lysis\ OD - background}$

Time-kill kinetics against bacteria and statistical analysis: The kinetic experiment was performed similarly as described in the minimum bactericidal concentration assay. MDR-*E. coli*-2 and MDR-*K. pneumoniae* bacteria were treated with PCo-MC. Mid-log phase (~10^8 CFU/mL) bacterial cell cultures were suspended to ~10^5 CFU/mL in media. 150 µL of this bacterial suspension was mixed with 50 µL of MIC/2, MIC, 2× MIC, and 4× MIC of the polymer followed by incubation at 37 °C overnight. 50 µL of water and imipenem antibiotic were used as negative and antibiotic control respectively. 20 µL samples of solutions from each concentration were then 10-fold serially diluted in 1× PBS at various time points such as 0, 20, 40, 60, 90, 120, 240,480, and 720 min. From these dilutions, 20 µL aliquots were spot plated on a tryptic agar plate and incubated for 24 h at 37 °C. After incubation, viable colonies were counted on the plates. These experiments were performed in triplicate and done twice on different times. For statistical analysis, log(CFU/mL) values were analyzed using a mixed-effects repeated-measures model including fixed effects for dose, time, and the dose × time interaction, with a random intercept to account for within replicate correlation across time. When the interaction indicated time-dependent treatment effects, time-specific pairwise comparisons among doses were conducted with Tukey adjustment for multiple testing.

Outer membrane permeabilization assay: Bacterial cultures were grown in TSB broth and incubated overnight at 37 °C in a shaking incubator. The bacterial culture was then centrifuged at 3500g for 5 minutes, and the supernatant was removed. The pellet was resuspended in 1× PBS and

subsequently suspended in a 1 : 1 mixture of 5 mM HEPES and 5 mM glucose buffer. *N*-Phenyl naphthylamine (NPN) dye was added to the bacterial suspension at a final concentration of 10 µM. A Corning black and clear bottom 96-well plate was prepared, with 180 µL of the prepared bacterial suspension added to each well. The fluorescence intensity of the NPN dye was measured at an excitation wavelength of 350 nm and an emission wavelength of 420 nm every 2 minutes. After 4 minutes, 20 µL of the test compound in water was added to the wells, with the final concentration adjusted using the microdilution method. The fluorescence intensity was recorded for an additional 24 minutes. Wells containing 20 µL of water instead of the compound served as negative controls. All experiments were performed in triplicate.

Membrane depolarization assay: Bacterial cultures were grown in TSB broth and incubated for 6 h at 37 °C in a shaking incubator to reach the mid-log phase. The bacterial culture was then centrifuged at 3500*g* for 5 minutes, and the supernatant was removed. The bacterial pellet was resuspended in 1× PBS and subsequently suspended in a 1 : 1 : 1 mixture of 5 mM HEPES buffer, 5 mM glucose, and 100 mM KCl solution and 0.2 mM ethylenediaminetetraacetic acid (EDTA) was added additionally for Gram-negative bacteria. $DiSC_3$ (3,3′-dipropylthiadicarbocyanine iodide) was added to the bacterial suspension to a final concentration of 2 µM, and the mixture was incubated in darkness for 1 hour. A Corning black and clear bottom 96-well plate was prepared with 180 µL of the bacterial suspension per well. The fluorescence intensity of the $DiSC_3$ dye was measured at an excitation wavelength of 622 nm and an emission wavelength of 670 nm every 2 minutes. After 4 minutes, 20 µL of the test compound in Millipore water was added, with the final concentration adjusted using the microdilution method. The fluorescence intensity was recorded for an additional 24 minutes. Wells containing 20 µL of Millipore water instead of the compound served as negative controls. All experiments were performed in triplicate.

LPS inhibition assay: The LPS inhibition assay was performed against MDR *E. hormaechei* and MDR *K. pneumoniae* according to previously reported method.(7) In a 96-well plate, 50 µL of polymer was 2-fold serially diluted from its highest concentration. 50 µL of LPS in normal saline was added to each well. After that, 100 µL of bacteria containing media was added to each well. The plate was incubated at 37 °C overnight and the optical density (OD_{600}) was measured. The positive and negative controls were, respectively, LPS and polymer incubated in the media. Each measurement was taken in triplicate.

Bacterial surface charge alteration: Bacteria cultures were grown in the TSB broth and incubated for 6 h at 37 °C to reach the mid-log phase. The bacterial culture was then centrifuged at 3500*g* for 5 minutes, and the supernatant was removed. The bacterial pellet was resuspended in 1× PBS for washing. After removing PBS, the bacterial pellet resuspended in normal saline. 1 mL of this bacterial solution was transferred to a sterile dry quartz cuvette, and the zeta potential was measured using a Zetasizer Nano. The measurement was taken in triplicate. Then polymer was adjusted to different concentration and added to the bacterial solution. Zeta potential was measured in triplicate.

Scanning electron microscopy (SEM): Briefly, 1 mL of ~10^5 CFU/mL mild-log phase bacteria were treated with polymer (32 µg/mL) in 1:1 mixture of Cation-adjusted Muller Hinton Broth (CMHB) and normal saline in a 6-well plate. In the case of control, bacterial cells were not treated

with the compound. Further, sterilized coverslips (tissue culture treated and 13 mm diameter) were added into each well with 2 mL bacterial suspension and incubated for 16 h at 37 °C under static conditions. The coverslips were taken out carefully into a new 6-well plate and were incubated with 2.5% glutaraldehyde solution in 0.1 M sodium cacodylate buffer for 2 hours at 4 °C. Next, the same coverslips were incubated in a 1% aqueous solution of osmium tetroxide for another hour at 4 °C and they were further washed with 0.1 M sodium cacodylate buffer to remove trace amount of toxic osmium tetroxide. Next, the coverslips were gradually incubated with 50%, 70%, 80%, 95% and 100% ethanol and each incubation step was for 10 min at room temperature. Finally, the coverslips (containing fixed and dehydrated bacterial cells) were dried using the critical point chamber, then coated with gold using a Denton Desk II sputter coater for 60 seconds, and images were captured using Zeiss Gemini500 FE-SEM. The images were further processed with ImageJ.

Biofilm eradication assay: The bacterial biofilms were grown by following the previously published protocol.(8) After the preparation of mid-log phase bacteria: MRSA and MDR-*P. aeruginosa* bacteria grown in TSB media, bacteria concentration was adjusted in NB with 1% (w/v) glucose and 1% (w/v) NaCl supplements to ~10^5 CFU/mL. Then the bacterial cells were inoculated in a tissue culture treated 96-well plate at 37 °C for 24 h (MRSA) and at 30 °C for 72 h (MDR *P. aeruginosa*). After the biofilm matured, the culture media was aspirated carefully. Followed by adding 200 µL of various concentrations of polymer and polymyxin-B antibiotic into the wells and incubated at 37 °C for 16-18 h. As a negative control, 200 µL of media was added without compound. The following three experiments were adjusted in the further steps as follows:

Quantification through Bacterial Cell Viability: The media was aspirated carefully and washed with 1× PBS to remove any remaining compound. Afterward, 200 µL of trypsin-EDTA solution (diluted in saline at a 1:4 ratio) was added to each well and incubated with shaking for 15 min. Samples of 20 µL of each concentration were then 10-fold serially diluted in 1× PBS. From these dilutions, 20 µL was spot plated on a tryptic soy agar plate and incubated for 20-24 h at 37 °C for counting viable colony forming units released from the biofilms. These experiments were performed in triplicate.

Crystal Violet Staining of Biofilm Biomass: Once again, the media was aspirated carefully and washed with 1× PBS to remove any remaining compound. Afterward, 0.1% of crystal violet (200 µL) dye was carefully added and left for 10 min for proper dying. The crystal violet was aspirated and stained wells were washed with 1 × PBS to remove excess crystal violet. The crystal violet stained biofilm containing wells were immediately dissolved with 95% ethanol. The absorbance of crystal violet containing ethanol was recorded at 570 nm. The absorbance of crystal violet dye was interpreted to correlate with the amount of biomass.

Fluorescence Microscopy of Biofilm Disruption: 18 mm glass coverslips were sterilized with flame after rinsing with ethanol. 2 mL of the diluted bacterial cell culture (~10^5 CFU/mL) was used to fill the 6-well plate and 18 mm sterilized glass coverslips were added to each well. The plate was incubated at 37 °C for 24 h (MRSA) and at 30 °C for 72 h (MDR *P. aeruginosa*). Coverslips were washed with 1× PBS and left to dry for 2 min. Biofilms were simultaneously stained with 5 µL of SYTO 9 (60 µM) and PI (15 µM) dye mixture and were transferred to a polylysine-coated Petri dish (MatTek Corporation) and imaged using a Zeiss

LSM700 confocal microscope in 63× magnification. Further, the images were processed by Image J- Fiji software.

Resistance study: The experiment was performed for PCo-MC against MDR *K. pneumoniae* and MDR *E. hormaechei*, with Polymyxin-B included for comparison for Gram-negative infections. MIC values were first determined using standard procedures. For subsequent passages, bacterial cultures were prepared from those exposed to sub-MIC (½ MIC) concentrations of the compound. This process was repeated for 22 consecutive passages. Resistance development was assessed by calculating changes in the MIC relative to the initial MIC and plotted over the passage number. All experiments were performed in triplicate.

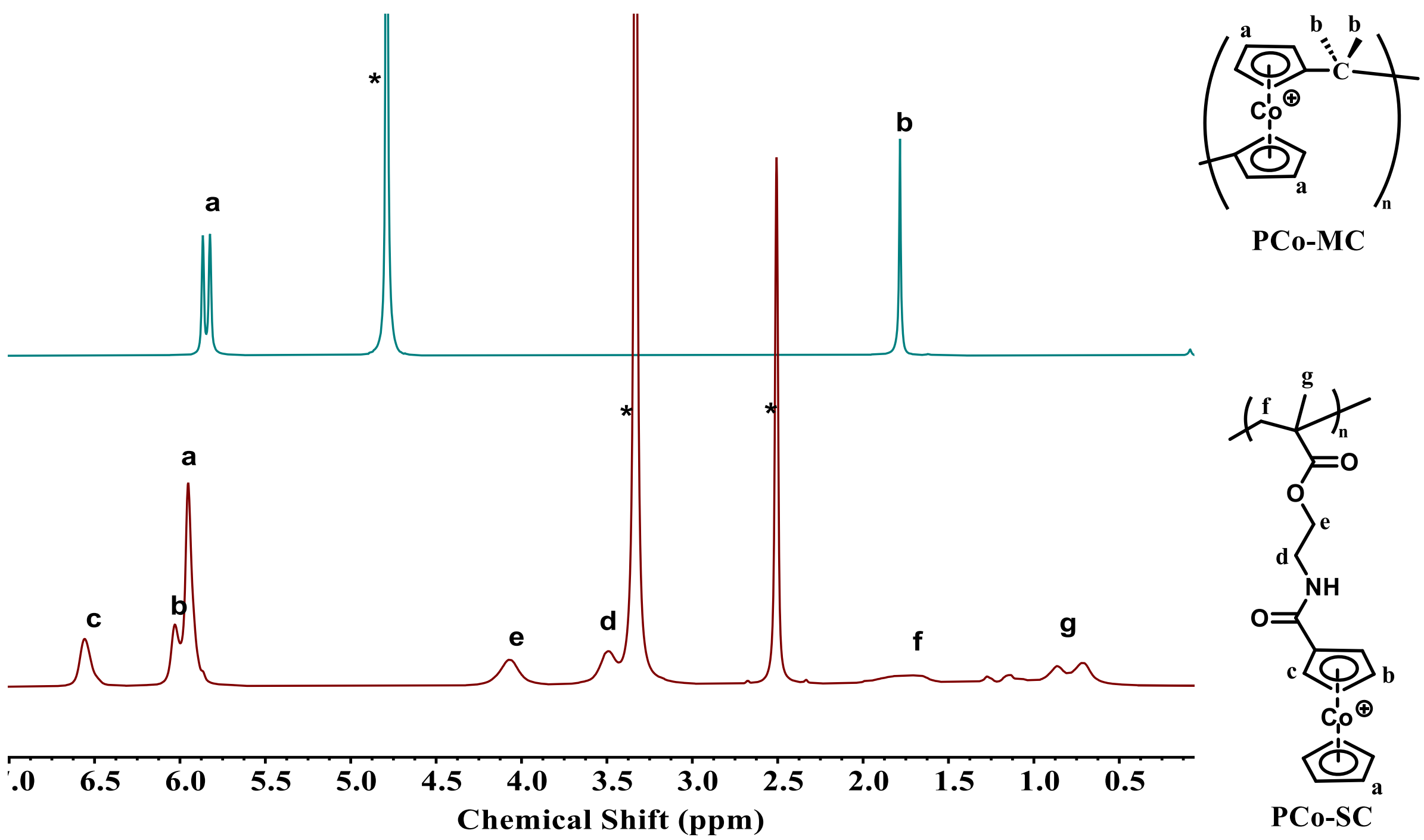

Figure S1. ^{1}H NMR spectra of **PCo-MC** (top) and **PCo-SC** (bottom).

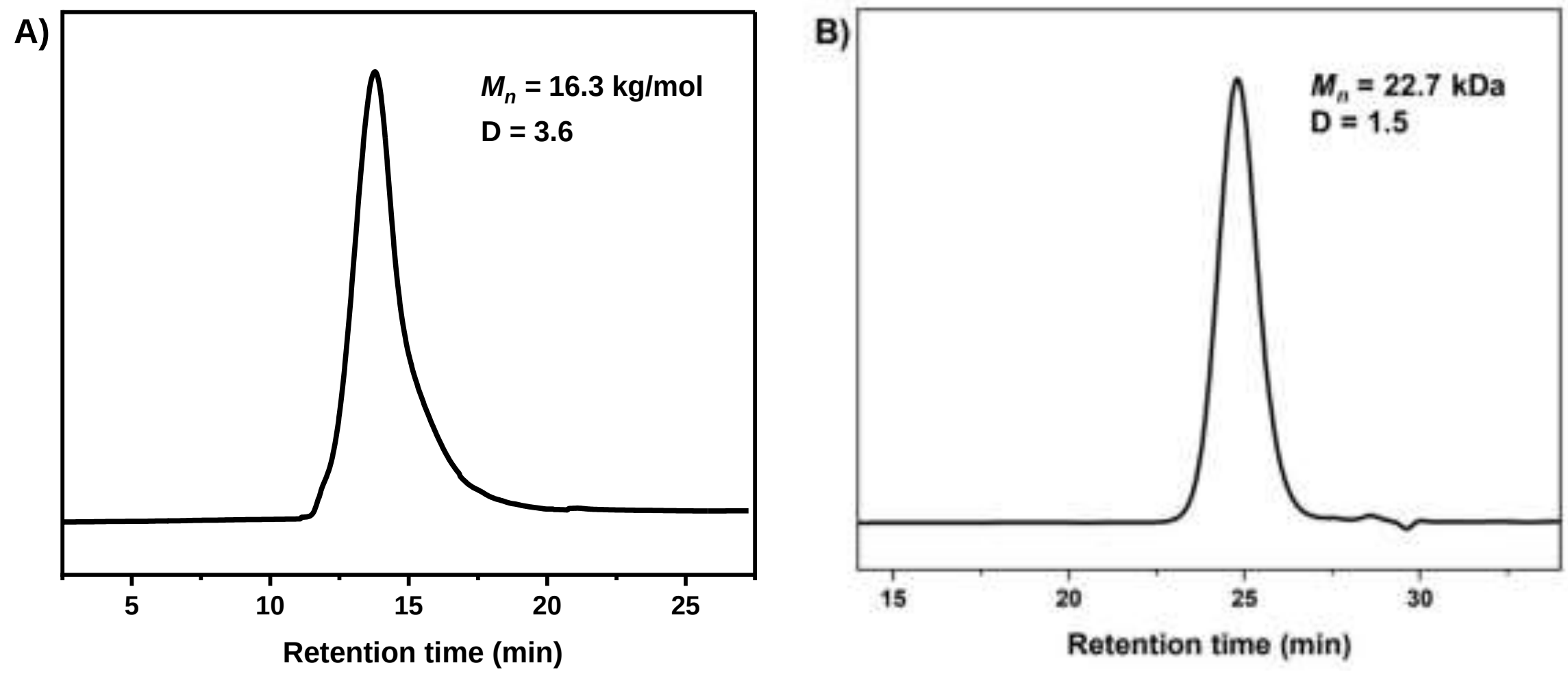

Figure S2: GPC trace of A) **PCo-MC** and B) **PCo-SC**.

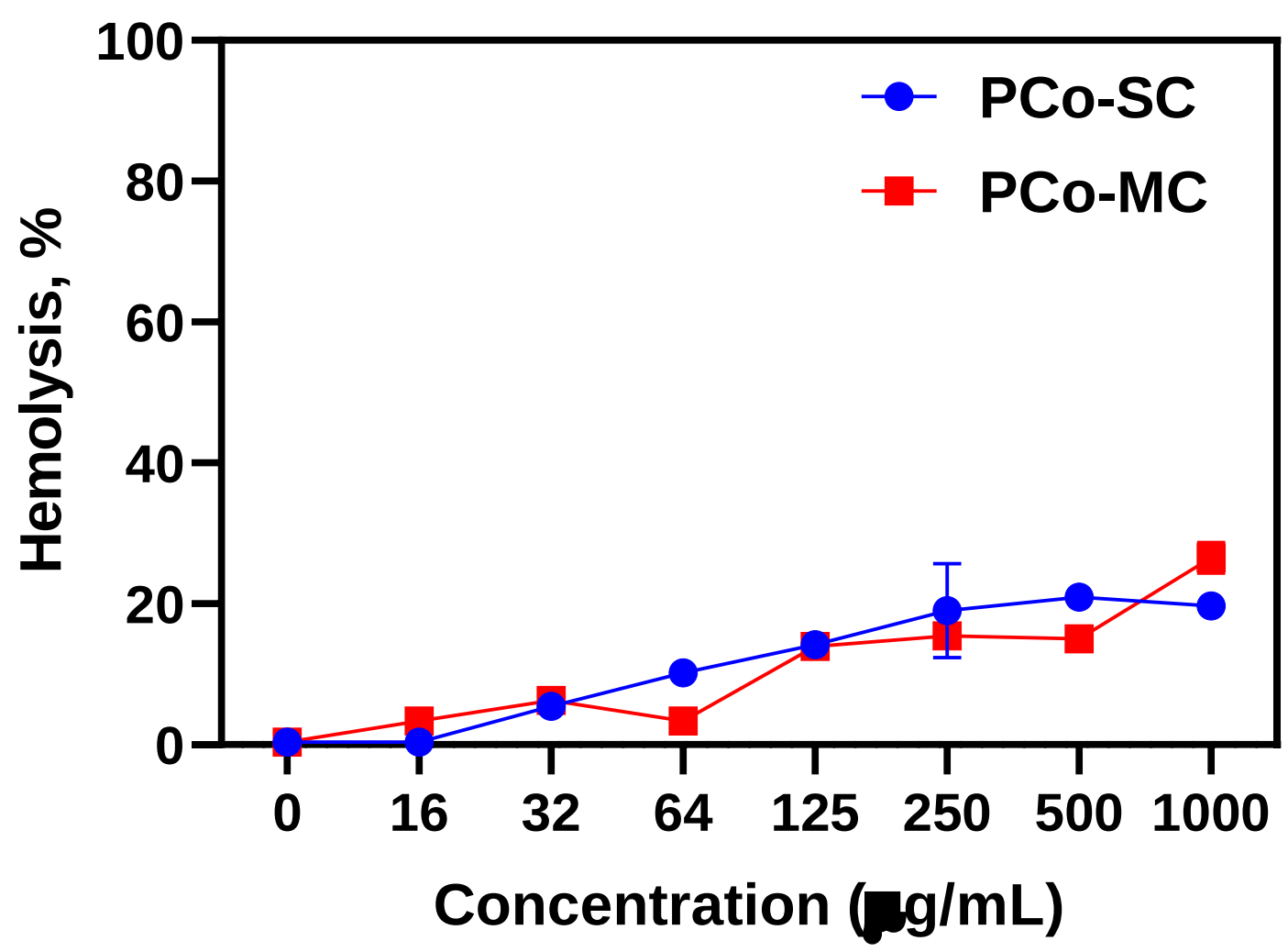

Figure S3: Hemolysis of mouse red blood cells (RBCs) by **PCo-MC** and **PCo-SC** at increasing concentration

Pairwise comparison of treatment groups at different time point for *E.coli*:

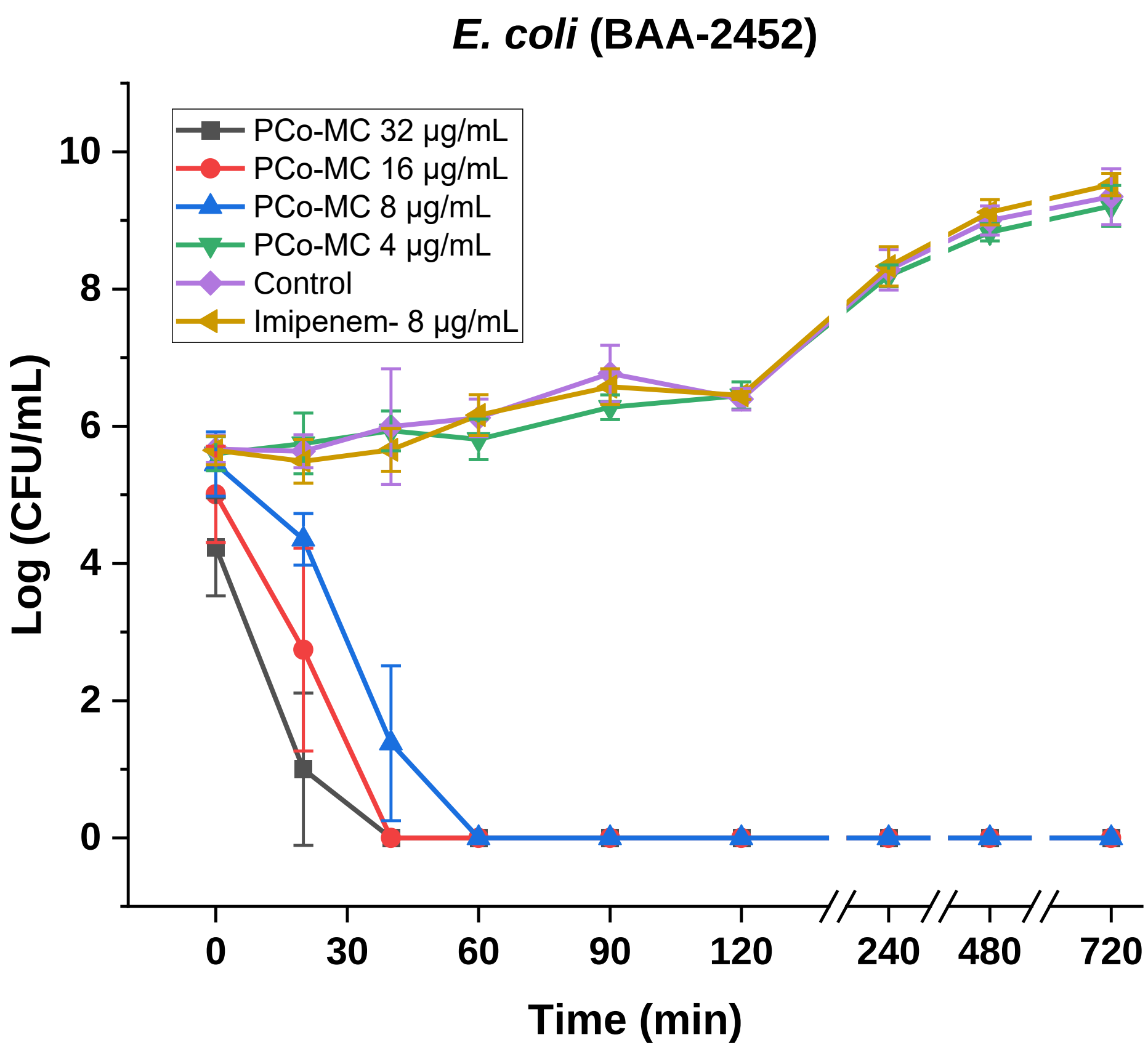

min = 0:

Contrast	Estimate	SE	df	t	*p*
Control - Imipenem 8	0.01866	0.231	260	0.081	1.0000
Control - (PCo-MC 16)	0.66139	0.231	260	2.868	0.0505
Control - (PCo-MC 32)	1.42674	0.231	260	6.186	<0.0001
Control - (PCo-MC 4)	0.06776	0.231	260	0.294	0.9997
Control - (PCo-MC 8)	0.21804	0.231	260	0.945	0.9342
Imipenem 8 - (PCo-MC 16)	0.64273	0.231	260	2.787	0.0628
Imipenem 8 - (PCo-MC 32)	1.40809	0.231	260	6.105	<0.0001
Imipenem 8 - (PCo-MC 4)	0.04910	0.231	260	0.213	0.9999
Imipenem 8 - (PCo-MC 8)	0.19938	0.231	260	0.865	0.9545
(PCo-MC 16) - (PCo-MC 32)	0.76535	0.231	260	3.318	0.0131
(PCo-MC 16) - (PCo-MC 4)	-0.59363	0.231	260	-2.574	0.1075
(PCo-MC 16) - (PCo-MC 8)	-0.44335	0.231	260	-1.922	0.3909

Contrast	Estimate	SE	df	t	*p*
(PCo-MC 32) - (PCo-MC 4)	-1.35899	0.231	260	-5.892	<0.0001
(PCo-MC 32) - (PCo-MC 8)	-1.20870	0.231	260	-5.241	<0.0001
(PCo-MC 4) - (PCo-MC 8)	0.15028	0.231	260	0.652	0.9868

min = 20:

Contrast	Estimate	SE	df	t	*p*
Control - Imipenem 8	0.14496	0.231	260	0.629	0.9888
Control - (PCo-MC 16)	2.89148	0.231	260	12.537	<0.0001
Control - (PCo-MC 32)	4.63490	0.231	260	20.096	<0.0001
Control - (PCo-MC 4)	-0.11352	0.231	260	-0.492	0.9964
Control - (PCo-MC 8)	1.28405	0.231	260	5.568	<0.0001
Imipenem 8 - (PCo-MC 16)	2.74652	0.231	260	11.909	<0.0001
Imipenem 8 - (PCo-MC 32)	4.48994	0.231	260	19.468	<0.0001
Imipenem 8 - (PCo-MC 4)	-0.25848	0.231	260	-1.121	0.8725
Imipenem 8 - (PCo-MC 8)	1.13909	0.231	260	4.939	<0.0001
(PCo-MC 16) - (PCo-MC 32)	1.74342	0.231	260	7.559	<0.0001
(PCo-MC 16) - (PCo-MC 4)	-3.00499	0.231	260	-13.029	<0.0001
(PCo-MC 16) - (PCo-MC 8)	-1.60743	0.231	260	-6.970	<0.0001
(PCo-MC 32) - (PCo-MC 4)	-4.74841	0.231	260	-20.589	<0.0001
(PCo-MC 32) - (PCo-MC 8)	-3.35085	0.231	260	-14.529	<0.0001
(PCo-MC 4) - (PCo-MC 8)	1.39757	0.231	260	6.060	<0.0001

min = 40:

Contrast	Estimate	SE	df	t	*p*
Control - Imipenem 8	0.33853	0.231	260	1.468	0.6851
Control - (PCo-MC 16)	5.99428	0.231	260	25.991	<0.0001
Control - (PCo-MC 32)	5.99428	0.231	260	25.991	<0.0001
Control - (PCo-MC 4)	0.06218	0.231	260	0.270	0.9998
Control - (PCo-MC 8)	4.61545	0.231	260	20.012	<0.0001

Contrast	Estimate	SE	df	t	*p*
Imipenem 8 - (PCo-MC 16)	5.65575	0.231	260	24.523	<0.0001
Imipenem 8 - (PCo-MC 32)	5.65575	0.231	260	24.523	<0.0001
Imipenem 8 - (PCo-MC 4)	-0.27635	0.231	260	-1.198	0.8375
Imipenem 8 - (PCo-MC 8)	4.27691	0.231	260	18.544	<0.0001
(PCo-MC 16) - (PCo-MC 32)	0.00000	0.231	260	0.000	1.0000
(PCo-MC 16) - (PCo-MC 4)	-5.93210	0.231	260	-25.721	<0.0001
(PCo-MC 16) - (PCo-MC 8)	-1.37883	0.231	260	-5.978	<0.0001
(PCo-MC 32) - (PCo-MC 4)	-5.93210	0.231	260	-25.721	<0.0001
(PCo-MC 32) - (PCo-MC 8)	-1.37883	0.231	260	-5.978	<0.0001
(PCo-MC 4) - (PCo-MC 8)	4.55327	0.231	260	19.742	<0.0001

min = 60:

Contrast	Estimate	SE	df	t	*p*
Control - Imipenem 8	-0.03871	0.231	260	-0.168	1.0000
Control - (PCo-MC 16)	6.12261	0.231	260	26.547	<0.0001
Control - (PCo-MC 32)	6.12261	0.231	260	26.547	<0.0001
Control - (PCo-MC 4)	0.31397	0.231	260	1.361	0.7500
Control - (PCo-MC 8)	6.12261	0.231	260	26.547	<0.0001
Imipenem 8 - (PCo-MC 16)	6.16132	0.231	260	26.715	<0.0001
Imipenem 8 - (PCo-MC 32)	6.16132	0.231	260	26.715	<0.0001
Imipenem 8 - (PCo-MC 4)	0.35268	0.231	260	1.529	0.6458
Imipenem 8 - (PCo-MC 8)	6.16132	0.231	260	26.715	<0.0001
(PCo-MC 16) - (PCo-MC 32)	0.00000	0.231	260	0.000	1.0000
(PCo-MC 16) - (PCo-MC 4)	-5.80864	0.231	260	-25.186	<0.0001
(PCo-MC 16) - (PCo-MC 8)	0.00000	0.231	260	0.000	1.0000
(PCo-MC 32) - (PCo-MC 4)	-5.80864	0.231	260	-25.186	<0.0001
(PCo-MC 32) - (PCo-MC 8)	0.00000	0.231	260	0.000	1.0000
(PCo-MC 4) - (PCo-MC 8)	5.80864	0.231	260	25.186	<0.0001

min = 90:

Contrast	Estimate	SE	df	t	*p*
Control - Imipenem 8	0.19062	0.231	260	0.827	0.9624
Control - (PCo-MC 16)	6.76757	0.231	260	29.343	<0.0001
Control - (PCo-MC 32)	6.76757	0.231	260	29.343	<0.0001
Control - (PCo-MC 4)	0.49017	0.231	260	2.125	0.2775
Control - (PCo-MC 8)	6.76757	0.231	260	29.343	<0.0001
Imipenem 8 - (PCo-MC 16)	6.57695	0.231	260	28.517	<0.0001
Imipenem 8 - (PCo-MC 32)	6.57695	0.231	260	28.517	<0.0001
Imipenem 8 - (PCo-MC 4)	0.29955	0.231	260	1.299	0.7855
Imipenem 8 - (PCo-MC 8)	6.57695	0.231	260	28.517	<0.0001
(PCo-MC 16) - (PCo-MC 32)	0.00000	0.231	260	0.000	1.0000
(PCo-MC 16) - (PCo-MC 4)	-6.27739	0.231	260	-27.218	<0.0001
(PCo-MC 16) - (PCo-MC 8)	0.00000	0.231	260	0.000	1.0000
(PCo-MC 32) - (PCo-MC 4)	-6.27739	0.231	260	-27.218	<0.0001
(PCo-MC 32) - (PCo-MC 8)	0.00000	0.231	260	0.000	1.0000
(PCo-MC 4) - (PCo-MC 8)	6.27739	0.231	260	27.218	<0.0001

min = 120:

Contrast	Estimate	SE	df	t	*p*
Control - Imipenem 8	-0.05493	0.231	260	-0.238	0.9999
Control - (PCo-MC 16)	6.39431	0.231	260	27.725	<0.0001
Control - (PCo-MC 32)	6.39431	0.231	260	27.725	<0.0001
Control - (PCo-MC 4)	-0.05346	0.231	260	-0.232	0.9999
Control - (PCo-MC 8)	6.39431	0.231	260	27.725	<0.0001
Imipenem 8 - (PCo-MC 16)	6.44924	0.231	260	27.963	<0.0001
Imipenem 8 - (PCo-MC 32)	6.44924	0.231	260	27.963	<0.0001
Imipenem 8 - (PCo-MC 4)	0.00146	0.231	260	0.006	1.0000
Imipenem 8 - (PCo-MC 8)	6.44924	0.231	260	27.963	<0.0001
(PCo-MC 16) - (PCo-MC 32)	0.00000	0.231	260	0.000	1.0000
(PCo-MC 16) - (PCo-MC 4)	-6.44777	0.231	260	-27.957	<0.0001
(PCo-MC 16) - (PCo-MC 8)	0.00000	0.231	260	0.000	1.0000
(PCo-MC 32) - (PCo-MC 4)	-6.44777	0.231	260	-27.957	<0.0001
(PCo-MC 32) - (PCo-MC 8)	0.00000	0.231	260	0.000	1.0000

Contrast	Estimate	SE	df	t	*p*
(PCo-MC 4) - (PCo-MC 8)	6.44777	0.231	260	27.957	<0.0001

min = 240:

Contrast	Estimate	SE	df	t	*p*
Control - Imipenem 8	-0.05047	0.231	260	-0.219	0.9999
Control - (PCo-MC 16)	8.27920	0.231	260	35.898	<0.0001
Control - (PCo-MC 32)	8.27920	0.231	260	35.898	<0.0001
Control - (PCo-MC 4)	0.08711	0.231	260	0.378	0.9990
Control - (PCo-MC 8)	8.27920	0.231	260	35.898	<0.0001
Imipenem 8 - (PCo-MC 16)	8.32967	0.231	260	36.117	<0.0001
Imipenem 8 - (PCo-MC 32)	8.32967	0.231	260	36.117	<0.0001
Imipenem 8 - (PCo-MC 4)	0.13759	0.231	260	0.597	0.9912
Imipenem 8 - (PCo-MC 8)	8.32967	0.231	260	36.117	<0.0001
(PCo-MC 16) - (PCo-MC 32)	0.00000	0.231	260	0.000	1.0000
(PCo-MC 16) - (PCo-MC 4)	-8.19208	0.231	260	-35.520	<0.0001
(PCo-MC 16) - (PCo-MC 8)	0.00000	0.231	260	0.000	1.0000
(PCo-MC 32) - (PCo-MC 4)	-8.19208	0.231	260	-35.520	<0.0001
(PCo-MC 32) - (PCo-MC 8)	0.00000	0.231	260	0.000	1.0000
(PCo-MC 4) - (PCo-MC 8)	8.19208	0.231	260	35.520	<0.0001

min = 480:

Contrast	Estimate	SE	df	t	*p*
Control - Imipenem 8	-0.12151	0.231	260	-0.527	0.9951
Control - (PCo-MC 16)	8.99878	0.231	260	39.018	<0.0001
Control - (PCo-MC 32)	8.99878	0.231	260	39.018	<0.0001
Control - (PCo-MC 4)	0.17159	0.231	260	0.744	0.9762
Control - (PCo-MC 8)	8.99878	0.231	260	39.018	<0.0001
Imipenem 8 - (PCo-MC 16)	9.12029	0.231	260	39.545	<0.0001
Imipenem 8 - (PCo-MC 32)	9.12029	0.231	260	39.545	<0.0001
Imipenem 8 - (PCo-MC 4)	0.29311	0.231	260	1.271	0.8006

Contrast	Estimate	SE	df	t	*p*
Imipenem 8 - (PCo-MC 8)	9.12029	0.231	260	39.545	<0.0001
(PCo-MC 16) - (PCo-MC 32)	0.00000	0.231	260	0.000	1.0000
(PCo-MC 16) - (PCo-MC 4)	-8.82718	0.231	260	-38.274	<0.0001
(PCo-MC 16) - (PCo-MC 8)	0.00000	0.231	260	0.000	1.0000
(PCo-MC 32) - (PCo-MC 4)	-8.82718	0.231	260	-38.274	<0.0001
(PCo-MC 32) - (PCo-MC 8)	0.00000	0.231	260	0.000	1.0000
(PCo-MC 4) - (PCo-MC 8)	8.82718	0.231	260	38.274	<0.0001

min = 720:

Contrast	Estimate	SE	df	t	*p*
Control - Imipenem 8	-0.17802	0.231	260	-0.772	0.9720
Control - (PCo-MC 16)	9.34566	0.231	260	40.522	<0.0001
Control - (PCo-MC 32)	9.34566	0.231	260	40.522	<0.0001
Control - (PCo-MC 4)	0.13414	0.231	260	0.582	0.9922
Control - (PCo-MC 8)	9.34566	0.231	260	40.522	<0.0001
Imipenem 8 - (PCo-MC 16)	9.52368	0.231	260	41.294	<0.0001
Imipenem 8 - (PCo-MC 32)	9.52368	0.231	260	41.294	<0.0001
Imipenem 8 - (PCo-MC 4)	0.31217	0.231	260	1.354	0.7546
Imipenem 8 - (PCo-MC 8)	9.52368	0.231	260	41.294	<0.0001
(PCo-MC 16) - (PCo-MC 32)	0.00000	0.231	260	0.000	1.0000
(PCo-MC 16) - (PCo-MC 4)	-9.21151	0.231	260	-39.940	<0.0001
(PCo-MC 16) - (PCo-MC 8)	0.00000	0.231	260	0.000	1.0000
(PCo-MC 32) - (PCo-MC 4)	-9.21151	0.231	260	-39.940	<0.0001
(PCo-MC 32) - (PCo-MC 8)	0.00000	0.231	260	0.000	1.0000
(PCo-MC 4) - (PCo-MC 8)	9.21151	0.231	260	39.940	<0.0001

Pairwise comparisons at each time for *K. pneumoniae*:

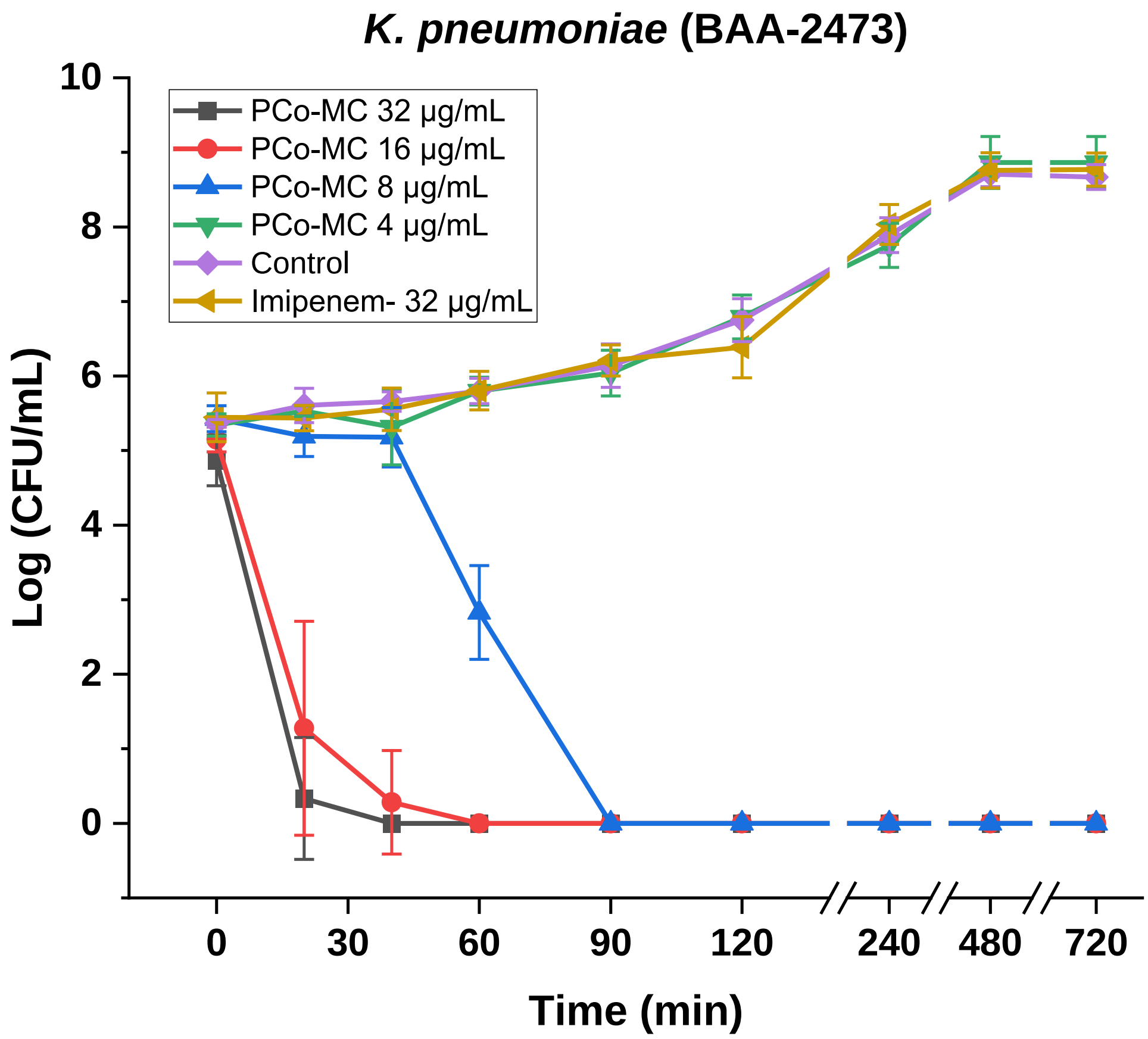

min = 0:

Contrast	Estimate	SE	df	t	*p*
Control - Imipenem 32	-0.079520	0.191	270	-0.417	0.9984
Control - (PCo-MC 16)	0.216814	0.191	270	1.137	0.8654
Control - (PCo-MC 32)	0.498254	0.191	270	2.614	0.0974
Control - (PCo-MC 4)	0.022406	0.191	270	0.118	1.0000
Control - (PCo-MC 8)	-0.060984	0.191	270	-0.320	0.9996
Imipenem 32 - (PCo-MC 16)	0.296334	0.191	270	1.555	0.6292
Imipenem 32 - (PCo-MC 32)	0.577775	0.191	270	3.031	0.0317
Imipenem 32 - (PCo-MC 4)	0.101926	0.191	270	0.535	0.9947
Imipenem 32 - (PCo-MC 8)	0.018537	0.191	270	0.097	1.0000
(PCo-MC 16) - (PCo-MC 32)	0.281440	0.191	270	1.476	0.6796
(PCo-MC 16) - (PCo-MC 4)	-0.194408	0.191	270	-1.020	0.9110
(PCo-MC 16) - (PCo-MC 8)	-0.277798	0.191	270	-1.457	0.6917
(PCo-MC 32) - (PCo-MC 4)	-0.475849	0.191	270	-2.496	0.1288

Contrast	Estimate	SE	df	t	*p*
(PCo-MC 32) - (PCo-MC 8)	-0.559238	0.191	270	-2.934	0.0419
(PCo-MC 4) - (PCo-MC 8)	-0.083389	0.191	270	-0.437	0.9980

min = 20:

Contrast	Estimate	SE	df	t	*p*
Control - Imipenem 32	0.169621	0.191	270	0.890	0.9487
Control - (PCo-MC 16)	4.330095	0.191	270	22.716	<0.0001
Control - (PCo-MC 32)	5.272297	0.191	270	27.659	<0.0001
Control - (PCo-MC 4)	0.076842	0.191	270	0.403	0.9986
Control - (PCo-MC 8)	0.414316	0.191	270	2.174	0.2536
Imipenem 32 - (PCo-MC 16)	4.160474	0.191	270	21.826	<0.0001
Imipenem 32 - (PCo-MC 32)	5.102676	0.191	270	26.769	<0.0001
Imipenem 32 - (PCo-MC 4)	-0.092780	0.191	270	-0.487	0.9966
Imipenem 32 - (PCo-MC 8)	0.244694	0.191	270	1.284	0.7938
(PCo-MC 16) - (PCo-MC 32)	0.942202	0.191	270	4.943	<0.0001
(PCo-MC 16) - (PCo-MC 4)	-4.253254	0.191	270	-22.313	<0.0001
(PCo-MC 16) - (PCo-MC 8)	-3.915779	0.191	270	-20.542	<0.0001
(PCo-MC 32) - (PCo-MC 4)	-5.195456	0.191	270	-27.255	<0.0001
(PCo-MC 32) - (PCo-MC 8)	-4.857981	0.191	270	-25.485	<0.0001
(PCo-MC 4) - (PCo-MC 8)	0.337474	0.191	270	1.770	0.4867

min = 40:

Contrast	Estimate	SE	df	t	*p*
Control - Imipenem 32	0.104478	0.191	270	0.548	0.9941
Control - (PCo-MC 16)	5.375314	0.191	270	28.199	<0.0001
Control - (PCo-MC 32)	5.658475	0.191	270	29.684	<0.0001
Control - (PCo-MC 4)	0.344138	0.191	270	1.805	0.4640
Control - (PCo-MC 8)	0.480927	0.191	270	2.523	0.1211
Imipenem 32 - (PCo-MC 16)	5.270836	0.191	270	27.651	<0.0001
Imipenem 32 - (PCo-MC 32)	5.553998	0.191	270	29.136	<0.0001
Imipenem 32 - (PCo-MC 4)	0.239660	0.191	270	1.257	0.8078
Imipenem 32 - (PCo-MC 8)	0.376450	0.191	270	1.975	0.3595
(PCo-MC 16) - (PCo-MC 32)	0.283162	0.191	270	1.485	0.6739

Contrast	Estimate	SE	df	t	*p*
(PCo-MC 16) - (PCo-MC 4)	-5.031176	0.191	270	-26.394	<0.0001
(PCo-MC 16) - (PCo-MC 8)	-4.894386	0.191	270	-25.676	<0.0001
(PCo-MC 32) - (PCo-MC 4)	-5.314338	0.191	270	-27.879	<0.0001
(PCo-MC 32) - (PCo-MC 8)	-5.177548	0.191	270	-27.161	<0.0001
(PCo-MC 4) - (PCo-MC 8)	0.136790	0.191	270	0.718	0.9797

min = 60:

Contrast	Estimate	SE	df	t	*p*
Control - Imipenem 32	-0.004825	0.191	270	-0.025	1.0000
Control - (PCo-MC 16)	5.799197	0.191	270	30.423	<0.0001
Control - (PCo-MC 32)	5.799197	0.191	270	30.423	<0.0001
Control - (PCo-MC 4)	0.000954	0.191	270	0.005	1.0000
Control - (PCo-MC 8)	2.970935	0.191	270	15.586	<0.0001
Imipenem 32 - (PCo-MC 16)	5.804022	0.191	270	30.448	<0.0001
Imipenem 32 - (PCo-MC 32)	5.804022	0.191	270	30.448	<0.0001
Imipenem 32 - (PCo-MC 4)	0.005779	0.191	270	0.030	1.0000
Imipenem 32 - (PCo-MC 8)	2.975760	0.191	270	15.611	<0.0001
(PCo-MC 16) - (PCo-MC 32)	0.000000	0.191	270	0.000	1.0000
(PCo-MC 16) - (PCo-MC 4)	-5.798243	0.191	270	-30.418	<0.0001
(PCo-MC 16) - (PCo-MC 8)	-2.828263	0.191	270	-14.837	<0.0001
(PCo-MC 32) - (PCo-MC 4)	-5.798243	0.191	270	-30.418	<0.0001
(PCo-MC 32) - (PCo-MC 8)	-2.828263	0.191	270	-14.837	<0.0001
(PCo-MC 4) - (PCo-MC 8)	2.969981	0.191	270	15.581	<0.0001

min = 90:

Contrast	Estimate	SE	df	t	*p*
Control - Imipenem 32	-0.070582	0.191	270	-0.370	0.9991
Control - (PCo-MC 16)	6.138290	0.191	270	32.202	<0.0001
Control - (PCo-MC 32)	6.138290	0.191	270	32.202	<0.0001
Control - (PCo-MC 4)	0.098511	0.191	270	0.517	0.9955
Control - (PCo-MC 8)	6.138290	0.191	270	32.202	<0.0001
Imipenem 32 - (PCo-MC 16)	6.208872	0.191	270	32.572	<0.0001

Contrast	Estimate	SE	df	t	*p*
Imipenem 32 - (PCo-MC 32)	6.208872	0.191	270	32.572	<0.0001
Imipenem 32 - (PCo-MC 4)	0.169093	0.191	270	0.887	0.9494
Imipenem 32 - (PCo-MC 8)	6.208872	0.191	270	32.572	<0.0001
(PCo-MC 16) - (PCo-MC 32)	0.000000	0.191	270	0.000	1.0000
(PCo-MC 16) - (PCo-MC 4)	-6.039779	0.191	270	-31.685	<0.0001
(PCo-MC 16) - (PCo-MC 8)	0.000000	0.191	270	0.000	1.0000
(PCo-MC 32) - (PCo-MC 4)	-6.039779	0.191	270	-31.685	<0.0001
(PCo-MC 32) - (PCo-MC 8)	0.000000	0.191	270	0.000	1.0000
(PCo-MC 4) - (PCo-MC 8)	6.039779	0.191	270	31.685	<0.0001

min = 120:

Contrast	Estimate	SE	df	t	*p*
Control - Imipenem 32	0.362018	0.191	270	1.899	0.4049
Control - (PCo-MC 16)	6.749537	0.191	270	35.408	<0.0001
Control - (PCo-MC 32)	6.749537	0.191	270	35.408	<0.0001
Control - (PCo-MC 4)	-0.042381	0.191	270	-0.222	0.9999
Control - (PCo-MC 8)	6.749537	0.191	270	35.408	<0.0001
Imipenem 32 - (PCo-MC 16)	6.387520	0.191	270	33.509	<0.0001
Imipenem 32 - (PCo-MC 32)	6.387520	0.191	270	33.509	<0.0001
Imipenem 32 - (PCo-MC 4)	-0.404399	0.191	270	-2.121	0.2793
Imipenem 32 - (PCo-MC 8)	6.387520	0.191	270	33.509	<0.0001
(PCo-MC 16) - (PCo-MC 32)	0.000000	0.191	270	0.000	1.0000
(PCo-MC 16) - (PCo-MC 4)	-6.791918	0.191	270	-35.630	<0.0001
(PCo-MC 16) - (PCo-MC 8)	0.000000	0.191	270	0.000	1.0000
(PCo-MC 32) - (PCo-MC 4)	-6.791918	0.191	270	-35.630	<0.0001
(PCo-MC 32) - (PCo-MC 8)	0.000000	0.191	270	0.000	1.0000
(PCo-MC 4) - (PCo-MC 8)	6.791918	0.191	270	35.630	<0.0001

min = 240:

Contrast	Estimate	SE	df	t	*p*
Control - Imipenem 32	-0.143271	0.191	270	-0.752	0.9751
Control - (PCo-MC 16)	7.889264	0.191	270	41.387	<0.0001

Contrast	Estimate	SE	df	t	*p*
Control - (PCo-MC 32)	7.889264	0.191	270	41.387	<0.0001
Control - (PCo-MC 4)	0.137641	0.191	270	0.722	0.9791
Control - (PCo-MC 8)	7.889264	0.191	270	41.387	<0.0001
Imipenem 32 - (PCo-MC 16)	8.032535	0.191	270	42.139	<0.0001
Imipenem 32 - (PCo-MC 32)	8.032535	0.191	270	42.139	<0.0001
Imipenem 32 - (PCo-MC 4)	0.280911	0.191	270	1.474	0.6814
Imipenem 32 - (PCo-MC 8)	8.032535	0.191	270	42.139	<0.0001
(PCo-MC 16) - (PCo-MC 32)	0.000000	0.191	270	0.000	1.0000
(PCo-MC 16) - (PCo-MC 4)	-7.751623	0.191	270	-40.665	<0.0001
(PCo-MC 16) - (PCo-MC 8)	0.000000	0.191	270	0.000	1.0000
(PCo-MC 32) - (PCo-MC 4)	-7.751623	0.191	270	-40.665	<0.0001
(PCo-MC 32) - (PCo-MC 8)	0.000000	0.191	270	0.000	1.0000
(PCo-MC 4) - (PCo-MC 8)	7.751623	0.191	270	40.665	<0.0001

min = 480:

Contrast	Estimate	SE	df	t	*p*
Control - Imipenem 32	-0.047276	0.191	270	-0.248	0.9999
Control - (PCo-MC 16)	8.709506	0.191	270	45.690	<0.0001
Control - (PCo-MC 32)	8.709506	0.191	270	45.690	<0.0001
Control - (PCo-MC 4)	-0.154076	0.191	270	-0.808	0.9658
Control - (PCo-MC 8)	8.709506	0.191	270	45.690	<0.0001
Imipenem 32 - (PCo-MC 16)	8.756782	0.191	270	45.938	<0.0001
Imipenem 32 - (PCo-MC 32)	8.756782	0.191	270	45.938	<0.0001
Imipenem 32 - (PCo-MC 4)	-0.106800	0.191	270	-0.560	0.9934
Imipenem 32 - (PCo-MC 8)	8.756782	0.191	270	45.938	<0.0001
(PCo-MC 16) - (PCo-MC 32)	0.000000	0.191	270	0.000	1.0000
(PCo-MC 16) - (PCo-MC 4)	-8.863581	0.191	270	-46.498	<0.0001
(PCo-MC 16) - (PCo-MC 8)	0.000000	0.191	270	0.000	1.0000
(PCo-MC 32) - (PCo-MC 4)	-8.863581	0.191	270	-46.498	<0.0001
(PCo-MC 32) - (PCo-MC 8)	0.000000	0.191	270	0.000	1.0000
(PCo-MC 4) - (PCo-MC 8)	8.863581	0.191	270	46.498	<0.0001

min = 720:

Contrast	Estimate	SE	df	t	*p*
Control - Imipenem 32	-0.100537	0.191	270	-0.527	0.9950
Control - (PCo-MC 16)	8.668865	0.191	270	45.477	<0.0001
Control - (PCo-MC 32)	8.668865	0.191	270	45.477	<0.0001
Control - (PCo-MC 4)	-0.194716	0.191	270	-1.021	0.9105
Control - (PCo-MC 8)	8.668865	0.191	270	45.477	<0.0001
Imipenem 32 - (PCo-MC 16)	8.769402	0.191	270	46.004	<0.0001
Imipenem 32 - (PCo-MC 32)	8.769402	0.191	270	46.004	<0.0001
Imipenem 32 - (PCo-MC 4)	-0.094179	0.191	270	-0.494	0.9964
Imipenem 32 - (PCo-MC 8)	8.769402	0.191	270	46.004	<0.0001
(PCo-MC 16) - (PCo-MC 32)	0.000000	0.191	270	0.000	1.0000
(PCo-MC 16) - (PCo-MC 4)	-8.863581	0.191	270	-46.498	<0.0001
(PCo-MC 16) - (PCo-MC 8)	0.000000	0.191	270	0.000	1.0000
(PCo-MC 32) - (PCo-MC 4)	-8.863581	0.191	270	-46.498	<0.0001
(PCo-MC 32) - (PCo-MC 8)	0.000000	0.191	270	0.000	1.0000
(PCo-MC 4) - (PCo-MC 8)	8.863581	0.191	270	46.498	<0.0001

References:

(1) Zhang, J.; Ren, L.; Hardy, C. G.; Tang, C. Cobaltocenium-Containing Methacrylate Homopolymers, Block Copolymers, and Heterobimetallic Polymers via RAFT Polymerization. *Macromolecules* **2012**, *45* (17), 6857-6863. DOI: 10.1021/ma3012784.
(2) Zhang, J.; Yan, Y.; Chance, M. W.; Chen, J.; Hayat, J.; Ma, S.; Tang, C. Charged Metallopolymers as Universal Precursors for Versatile Cobalt Materials. *Angewandte Chemie International Edition* **2013**, *52* (50), 13387-13391. DOI: https://doi.org/10.1002/anie.201306432.
(3) Wirtz, L.; Haider, W.; Huch, V.; Zimmer, M.; Schäfer, A. Magnesocenophane-Catalyzed Amine Borane Dehydrocoupling. *Chemistry–A European Journal* **2020**, *26* (28), 6176-6184.
(4) Cremer, C.; Jacobsen, H.; Burger, P. The Metal Oxo [1, 2]-Alkyl Shift–Towards the Aerobic Oxidation of Hydrocarbons. *Chimia* **1997**, *51* (8-9), 650-653.
(5) Perrotin, P.; Shapiro, P. J.; Williams, M.; Twamley, B. In Search of a Versatile Pathway to ansa-Chromocene Complexes. Synthesis and Characterization of the Highly Unstable ansa-Chromocene Carbonyl Complex Me2C (C5H4) 2CrCO. *Organometallics* **2007**, *26* (7), 1823-1826.
(6) Kurnaz, L. B.; Barman, S.; Yang, X.; Fisher, C.; Outten, F. W.; Nagarkatti, P.; Nagarkatti, M.; Tang, C. Facial amphiphilic naphthoic acid-derived antimicrobial polymers against multi-drug resistant gram-negative bacteria and biofilms. *Biomaterials* **2023**, *301*, 122275.
(7) Hossain, M. W.; Brand, I.; Barman, S.; Abidoun, A.; Hwang, J.; Parris, A.; Yang, X.; Nagarkatti, P.; Nagarkatti, M.; Tang, C. Antimicrobial cobaltocenium copolymers: tuning

amphiphilicity against NDM-1 bacteria. *Biomaterials Science* **2025**, *13* (15), 4232-4244. DOI: 10.1039/d5bm00497g.
(8) Qian, Y.; Deng, S.; Cong, Z.; Zhang, H.; Lu, Z.; Shao, N.; Bhatti, S. A.; Zhou, C.; Cheng, J.; Gellman, S. H.; Liu, R. Secondary Amine Pendant β-Peptide Polymers Displaying Potent Antibacterial Activity and Promising Therapeutic Potential in Treating MRSA-Induced Wound Infections and Keratitis. *Journal of the American Chemical Society* **2022**, *144* (4), 1690-1699. DOI: 10.1021/jacs.1c10659.
